# *Salvia pratensis* exhibits *in vitro* anti-cancer effects in triple-negative breast cancer through miR-34a-5p signaling

**DOI:** 10.3389/fnut.2026.1786148

**Published:** 2026-03-18

**Authors:** Clarissa Gervasoni, Chiara Ceriani, Aurora Lanzotti, Stefano Negri, Flavia Guzzo, Fabio Pirovano, Bruno Giovanni Galuzzi, Francesca Annè, Alessandra Inguscio, Alessia Lo Dico, Danilo Porro, Gloria Rita Bertoli

**Affiliations:** 1 Institute of Bioimaging and Complex Biological Systems - National Research Council (IBSBC-CNR), Segrate, Italy; 2 National Biodiversity Future Center (NBFC), Palermo, Italy; 3 Department of Earth and Marine Sciences (DiSTeM), University of Palermo, Palermo, Italy; 4 Department of Biotechnology, University of Verona, Verona, Italy; 5 Department of Biotechnology and Biosciences, University of Milano-Bicocca, Milan, Italy

**Keywords:** apoptosis, MicroRNA, nature-based solution, oxidative stress, triple-negative breast cancer, MDA-MB-231

## Abstract

**Introduction:**

Triple-negative breast cancer (TNBC) is the most aggressive malignancy in women, with limited treatment options and significant adverse effects. Natural products have emerged as promising anticancer agents, either alone or in combination with standard therapies. Within the National Biodiversity Future Center, this study aims to valorize Italy’s plant biodiversity, as a source of novel bioactive compounds with therapeutic and nutraceutical potential.

**Methods:**

Leaf extracts of *Petasites paradoxus*, *Salvia pratensis*, and *Typha laxmannii* were profiled by UPLC-ESI-HRMS. TNBC MDA-MB-231 and non-tumorigenic MCF 10A mammary epithelial cells were exposed to 5 μg/mL of each DMSO-resuspended extract. Viability, proliferation, migration, cell-cycle, and ROS were assessed. Genes and miRNA expression were quantified by RT-qPCR, supported by immunofluorescence, bioinformatic functional enrichment, and patients’ survival analyses.

**Results:**

Each species showed a specific phytochemical fingerprint. Notably, *S. pratensis*, enriched in rosmarinic acid, caffeic acid esters, and luteolin derivatives, selectively reduced TNBC cell viability after 24 h. The treatment also impaired proliferation, migration, and cell cycle, increasing mitochondrial reactive oxygen species, and inducing apoptosis. Underlying the molecular mechanisms, *S. pratensis* stimulation modulated genes associated with tumor aggressiveness. Importantly, over-representation analysis on the differentially expressed genes pool identified miR-34a-5p as a potential regulator, its low expression being linked to poor TNBC prognosis. MiR-34a-5p in treated MDA-MB-231 was significantly up-regulated compared to controls.

**Discussion:**

These findings position *S. pratensis* as a promising source of naturally derived bioactive compounds with selective anticancer activity, mediated in part via miR-34a-5p signaling. This study highlights the potential of plant-based bioactives for preventive and complementary strategies in TNBC, supporting further exploration of their integration into dietary or therapeutic interventions.

## 1 Introduction

Triple-negative breast cancer (TNBC) is the most common and aggressive type of diagnosed malignancies in women. It accounts for approximately 15% of newly diagnosed breast cancer cases and contributes to around 5% of all cancer-related deaths (1). TNBC is defined by the absence of estrogen receptor (ER), progesterone receptor (PR), and human epidermal growth factor receptor 2 (HER2) expression. The lack of conventional therapeutic targets makes TNBC particularly difficult to treat and is still associated with poor prognosis (2). The first-line treatments for early-stage and localized TNBC typically include neoadjuvant chemotherapy (e.g., anthracyclines and taxanes), radiation and surgery (3). Instead, for patients diagnosed with metastatic TNBC, the standard therapy focuses on a combination of immunotherapy and chemotherapy, with surgical and radiotherapeutic integrations. All current treatments are associated with a high risk of drug resistance and have a drastic impact on patients’ quality of life. Unfortunately, the aggressiveness and heterogeneity of TNBC result in a higher risk of relapse within the first 3–5 years compared to other breast cancer subtypes and are frequently associated with severe drug resistance. As a result, the median overall survival for early-stage TNBC is currently approximately 10 months, with a 5-year survival rate of ~65% for cases involving regional spread. However, this rate drastically drops to about 10% in metastatic TNBC (1, 4). Despite significant therapeutic advances in recent years, TNBC remains a critical and challenging disease.

Emerging studies have demonstrated the anticancer properties of naturally derived bioactive molecules. The metabolites naturally present in fruits, vegetables, herbs, oils, and other plant-derived foods have emerged as key regulators of oxidative stress, inflammation, apoptosis, and cellular homeostasis, processes that are central to cancer biology. Importantly, approximately half of currently approved anticancer drugs originate from natural sources, underlying the importance of bioactive compounds as reservoirs of pharmacologically active molecules (5–7). Despite ongoing efforts to develop effective treatments with minimal side effects, therapeutic options remain limited to a narrow window with significant toxicity.

The National Biodiversity Future Center (NBFC)^1^ is committed to monitoring, promoting, and valorizing the Italian biodiversity, offering a promising avenue to discover novel therapeutic compounds and to isolate them by biotechnology application (8). Within this framework, our project aims to explore selected components of Italian flora as sources of food-derived and food-inspired bioactive molecules with potential relevance for cancer prevention and complementary therapeutic strategies. In particular, we focused on *Petasites paradoxus (Retz.) Baumg., Salvia pratensis L.*, and *Typha laxmannii Lepech*, plant species characterized by documented ethnobotanical use and phytochemical richness, and traditionally consumed or employed in edible preparations, herbal infusions, or functional plant-based products (9–11). The hypothesis of this study was to deeply characterize the potential anticancer effects of these extracts, proposing that either the whole extracts or the bioactive molecules within them could reduce TNBC aggressiveness. Specifically, we evaluated the effects of selected natural-based extracts on an *in vitro* TNBC model, characterized by its high aggressiveness and invasiveness, focusing on their impact on cellular and molecular mechanisms. This approach holds promise for addressing the therapeutic challenges posed by TNBC and may pave the way for new pharmacological strategies.

## 2 Materials and methods

### 2.1 Plant extract preparation and UPLC-ESI-HRMS analysis

Leaves of *Petasites paradoxus (Retz.) Baumg., Salvia pratensis L.*, and *Typha laxmannii Lepech.* were collected from the institutional nursery of Veneto Agricoltura (Vicenza, Italy). Extract preparation and processing followed the procedures detailed in Supplementary methods.

Methanolic extracts were analyzed by UPLC–ESI–HRMS (Acquity I-Class UPLC coupled to Xevo G2-XS qTOF, Waters) in positive and negative ionization modes under reverse-phase (C18) conditions, as described by Giammona *et al.* (12). Metabolites were identified based on accurate mass, isotopic pattern, retention time, and fragmentation spectra, through comparison with authentic standards, proprietary and in silico libraries, and public databases (MassBank, MoNA).

### 2.2 Cell culture, treatments, and viability assays

Human triple-negative breast cancer (MDA-MB-231) and non-tumorigenic epithelial breast (MCF 10A) cell lines were maintained under standard culture conditions (37 °C, 5% CO₂). Cells were treated for 24 h and 48 h with methanolic extracts of *P. paradoxus*, *S. pratensis*, and *T. laxmannii* (5 μg/mL) or with vehicle control (DMSO, 1% v/v). Cell viability and proliferation were assessed using MTT (Promega, Madison, WI, USA) and CellTiter-Glo® assays according to the manufacturer’s instructions. All experimental procedures are reported in Supplementary methods.

### 2.3 Migration assays

Cell migration was evaluated using scratch and transwell (Boyden chamber) assays. For the scratch assay, MDA-MB-231 cells were seeded in 24-well plates and, after wound creation, treated with *S. pratensis* extract or vehicle (DMSO). Images were acquired at 0, 6, 24, and 30 h, and wound closure was quantified by ImageJ software. For the Boyden chamber assay, cells were seeded and allowed to migrate toward serum-containing media for 24 h. Non-migrated cells were removed, and migrated cells were stained and quantified using ImageJ. Detailed experimental conditions are reported in Supplementary methods.

### 2.4 Cell cycle analysis

Cell cycle was evaluated by flow cytometry after propidium iodide staining. Cells were fixed in cold ethanol, stained, and analyzed on a FACS Celesta cytometer (BD Life Sciences). Data were processed with FlowJo Software v.10. Detailed procedures for fixation, staining, and acquisition are provided in Supplementary methods.

### 2.5 Mitochondrial ROS evaluation by MitoSOX® assay

ROS production was quantified using MitoSOX® Red Mitochondrial Superoxide Indicator assay (Invitrogen, Thermo Fisher Scientific), following the manufacturer’s instructions. The samples were analyzed using a FACS Celesta flow cytometer (BD Biosciences), and the obtained data were processed with FlowJo Software.

### 2.6 Gene expression analysis by real-time quantitative PCR (RT-qPCR)

Total RNA extraction, cDNA synthesis, and quantitative PCR for gene and miRNA expression were performed as described in Supplementary methods. Briefly, total RNA was isolated from MDA-MB-231 cells using TRIzol™ reagent (Thermo Fisher Scientific), reverse transcribed, and analyzed by real-time PCR using SYBR™ Green chemistry on a CFX Connect system (Bio-Rad). Relative expression levels were calculated using the 2^−^ΔΔCt method (13), normalized to 14S (genes) and U6 (miRNAs).

### 2.7 Immunofluorescence analysis

Immunofluorescence staining of BAX and BCL2 was performed on fixed cells treated with *S. pratensis* extract, or vehicle for 24 h. Cells were processed, labeled with specific primary and fluorescent secondary antibodies, and imaged using a Stellaris confocal microscope (Leica). Signal quantification was carried out with Fiji software. Detailed antibody information and imaging conditions are reported in Supplementary methods.

### 2.8 Over-representation analysis

Over-representation analysis of differentially expressed genes and miRNA–mRNA interactions was conducted as described in Supplementary methods. Briefly, over-representation of Gene Ontology terms was assessed using the ShinyGO tool (14). Enrichment of experimentally validated miRNA targets was evaluated using DIANA-TarBase (15) and miRTarBase (16), focusing on miRNAs with established tumor-suppressive roles in cancer pathophysiology.

### 2.9 Kaplan–Meier survival analysis

Survival analysis based on miRNA expression was performed using the Kaplan–Meier plotter tool (17). Patient cohorts were stratified according to expression levels of selected miRNAs, and relapse-free and overall survival were analyzed using Kaplan–Meier curves, with hazard ratios (95% CI) and log-rank *p*-values calculated automatically. Detailed procedures and inclusion criteria are provided in Supplementary methods.

### 2.10 Transfection

MDA-MB-231 cells were pre-seeded in 12-well plates and transfected at 70% confluence, with miR-34a-5p mimic or a scrambled miRNA control (Supplementary Table S2) at two concentrations (dose 1: 0.6 μg/mL; dose 2: 0.8 μg/mL). Transfections were performed using Attractene Transfection Reagent (Qiagen, Hilden, Germany), according to the manufacturer’s instructions. Briefly, miRNA mimic or scramble control was diluted in 80 μL of serum- and antibiotics-free medium, followed by the addition of Attractene Reagent. The mixture was incubated at room temperature for 20 min to allow formation of transfection complexes. Subsequently, 80 μL of the complexes was added dropwise to the cells and after 24 h, cells were harvested for the proliferation assay.

### 2.11 Statistical data analysis

Normal distribution was evaluated by the Shapiro–Wilk test before performing parametric or non-parametric tests in each experiment. The statistical analysis was performed using Mann–Whitney t-test or one-way ANOVA test in Prism 7. For the ORA analysis, hypergeometric testing and Benjamini–Hochberg correction (FDR < 0.05) were applied. For the survival analysis, statistical differences between groups were assessed using the log-rank test. A *p* < 0.05 was considered statistically significant.

## 3 Results

### 3.1 Phytochemical characterization of plant extracts

**Figure 1:**
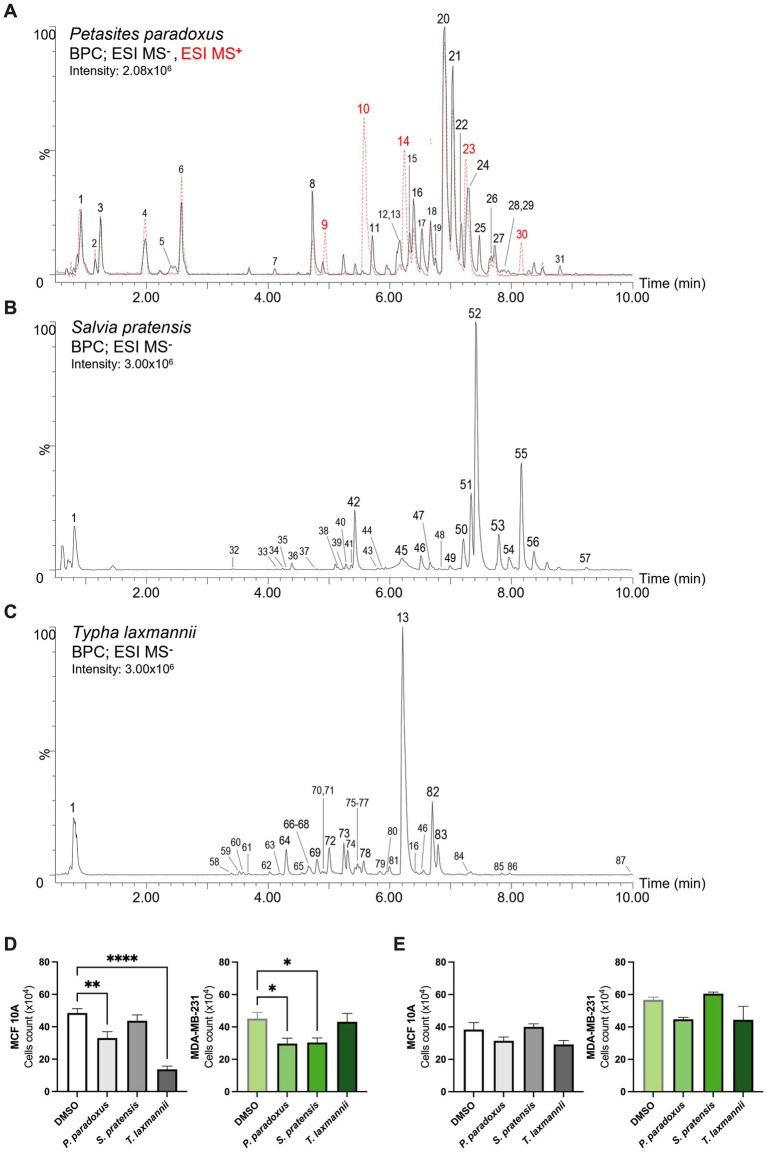
Natural extracts characterization and effects on MCF 10A and MDA-MB-231 cell proliferation. **(A–C)** Base peak chromatograms (BPC) obtained from the metabolomic analysis of *Petasites paradoxus* (ESI-; ESI+), *Salvia pratensis* (ESI-) and *Typha laxmannii* (ESI-) extracts. Peak numbers refer to metabolites and LC–MS features listed in Supplementary Table S1. **(D,E)** Cell proliferation assessed by cell counts after 24 h (left panel) and 48 h (right panel) of treatments (*n* ≥ 6; one-way ANOVA followed by Dunnett’s multiple comparisons test). Data are presented as mean ± SEM. *p < 0.05; **p < 0.01; ****p < 0.0001.

The untargeted metabolomics analysis performed using UPLC-ESI-HRMS revealed distinct phytochemical profiles among the three species (Figures 1A–C). All detailed LC–MS features of the principal metabolites identified in the methanolic leaf extracts are listed in Supplementary Table S1.

#### 3.1.1 Petasites paradoxus

The negative ionization analysis of *Petasites paradoxus (P. paradoxus)* revealed a phytochemical profile mainly characterized by high levels of hydroxycinnamic acid derivatives, flavonols and oligosaccharides. (Figure 1A) Within the hydroxycinnamic acid derivatives, the most abundant compounds were an isomer of dicaffeoylquinic acid [20], 3-O-caffeoylquinic acid [chlorogenic acid, 8] and fukinolic acid [17], which is an ester of caffeic acid and fukiic acid ((3,4-dihydroxybenzyl)tartaric acid), rarely reported in plants but already described in *Petasites* (18). At lower levels, also various caffeoylferuloylquinic acid isomers [26–29], other dicaffeoylquinic acid isomers [19, 22] and 5-O-caffeoylquinic acid [neochlorogenic acid, 7] were detected. Among flavonols, a wide array of quercetin glycosides was identified, including quercetin 3-O-galactoside [hyperoside, 15] and quercetin 3-O-glucoside [isoquercetin, 16], as well as acylated forms including two isomers of quercetin-O-(O-acetyl)-hexoside [18, 21] and quercetin-O-(O-caffeoyl)-hexoside [24]. In the polar range of the analysis, several oligosaccharides with varying degrees of polymerization [ranging from tri- to octa-hexoses; 2–6] were detected.

Additional characteristic metabolites included the sulfated sesquiterpene fukinoside A [31], previously found in *P. japonicus* (19), and its dehydrated form, dehydrofukinoside A [25]. In positive ionization mode, different otonecine-type pyrrolizidine alkaloids previously described in the genus *Petasites* (11) were detected. These included fukinotoxin [petasitenine, 10] and senkirkine [14], showing the fragmentation patterns described by Kitajima et al. (20), together with their acetylated derivatives neopetasitenine [23] and acetylsenkirkine [30].

#### 3.1.2 Salvia pratensis

The leaf extract of *Salvia pratensis (S. pratensis)*, analyzed in negative ionization mode (Figure 1B), was strongly characterized by the accumulation of various caffeic acid esters, which are important chemotaxonomic markers of Lamiaceae family (21). Rosmarinic acid [52], the ester of caffeic acid and 3,4-dihydroxyphenyllactic acid [danshensu or salvianic acid A, 32], was the major constituent of the extract while several of its derivatives were detected, including rosmarinic acid hydrate [Danshensuan C, 42], methylrosmarinate [56], two O-hexosides [47, 48] and two unidentified compounds [43, 44] exhibiting a similar fragmentation pattern. Other compounds structurally related to rosmarinic acid included the salvianolic acids A, K and L [45, 51, 57], a lithospermic acid isomer [53] and the cyclobutane lignan sagerinic acid [49], all previously described in *Salvia* spp. (22). Other hydroxycinnamic acid derivatives included isomeric esters of caffeic or ferulic acid with threonic acid [33, 35, 36, 38, 41]. The flavonoid profile is consistent with those typically observed in sage species, where luteolin derivatives are often dominant (22). A luteolin-O-(O-acetyl)-hexoside [50] represented the main compound in this class, followed by luteolin-7-O-glucoside [cynaroside, 46] and a luteolin-methoxylated derivative tentatively identified as chrysoeriol-O-hexoside carrying a hydroxymethylglutaryl moiety. The second most abundant metabolite present in the extract was tentatively identified as the O-(O-pentosyl)-hexoside of oct-3-en-1-ol [55], which is a fatty alcohol found within the essential oils of various Lamiaceae species (23). At lower levels, several other glycosides of different compounds were detected in the extract including those of coumaric [37] and syringic acid [34], a benzyl alcohol-(O-pentosyl)-hexoside [39] and the O-hexoside of the jasmonate-related metabolite tuberonic acid [40].

#### 3.1.3 Typha laxmannii

The methanolic leaf extract of *Typha laxmannii (T. laxmannii)* analysed in negative ionization mode revealed a profile (Figure 1C) mainly characterized by the accumulation of flavanol glycosides and, at lower level, a broad range of flavan-3-ols oligomers (proanthocyanidins). In particular, the most abundant compounds in the extracts were the 3-O-rutinosides of quercetin [rutin, 13], kaempferol [nictoflorin, 82] and isorhamnetin [narcissoside, 83]. Additional flavonoid glycosides present at lower levels included quercetin-3-O-glucoside [isoquercetin, 16], luteolin-7-O-glucoside [cynaroside, 46], apigenin 6,8-di-C-glucoside [vicenin-2, 73] and another di-C-glucoside of a flavanone tentatively identified as eriodictyol [68]. Flavan-3-ols such as catechin [67], epicatechin [74], gallocatechin [59] and (epi)afzelechin [80] were detected giving rise to a wide range of structurally diverse B-type proanthocyanidins. These were mainly represented by (epi)catechin-(epi)catechin dimers [procyanidins; 63–65], including procyanidin B2 [72], (epi)gallocatechin-(epi)catechin dimers [prodelphinidins; 58, 61, 62] and (epi)afzelechin-(epi)catechin dimers [propelargonidins; 69, 75, 78, 81]. High-order oligomers such as the trimeric Procyanidin C1 [77] and one trimer involving one (epi)afzelechin and two (epi)catechin units [79] were also detected, although in lower amounts.

Hydroxycinnamic acid derivatives were mainly represented by esters of caffeic acid, including caffeoylshikimic acid [76] and its hexoside [71], and two of its sulfated derivatives [60, 70]. Additional low-abundance compounds in this class included two unidentified compounds carrying sinapoyl [85] and feruloyl [86] moieties and coumaroylferuloyl glycerol [87].

### 3.2 Effects of natural extracts on MCF 10A and MDA-MB-231 cell viability

To identify natural-derived biomolecules with selective cytotoxic activity, we stimulated the TNBC cell line MDA-MB-231 and the non-tumorigenic mammary epithelial cell line MCF 10A with plant-derived extracts from *P. paradoxus*, *S. pratensis*, and *T. laxmannii* for 24 and 48 h at the final concentration of 5 μg/mL; previous analyses revealed that this is the effective concentration for TNBC cell lines (unpublished data). Notably, *S. pratensis* significantly impaired the MDA-MB-231 viability by 33% after 24 h of treatment compared to controls condition, without affecting MCF 10A cells’ count. Conversely, we demonstrated that 24 h of treatment with *P. paradoxus* and *T. laxmannii* significantly reduced the MCF 10A cells number by 32% (*p* = 0.004) and 72% (*p* < 0.0001), respectively (Figure 1D). No significant effects were observed at 48 h in either cell line (Figure 1E). Based on these results, we further investigated *S. pratensis* effects as potential anticancer candidate, and the experiments conducted afterwards were focused solely on this species.

### 3.3 *Salvia pratensis* reduced the metabolic activity of MDA-MB-231 with high mitochondrial ROS and cell cycle arrest

**Figure 2:**
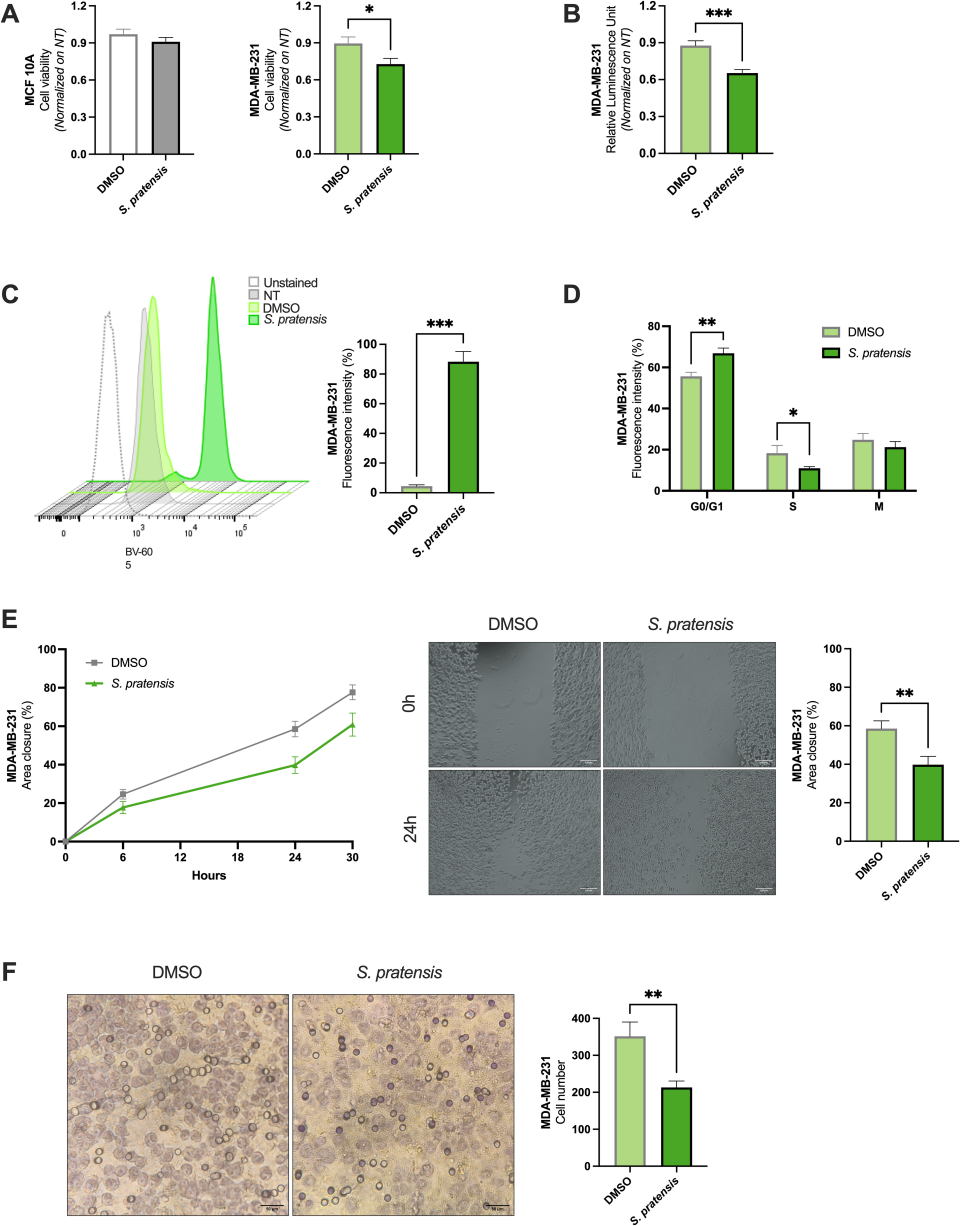
Effects of *S. pratensis* extract on cell viability, oxidative stress, cell cycle distribution, proliferation, and migration. **(A)** Cell viability measured by MTT assay, expressed as mean absorbance values after 24 h of treatment, normalized on untreated cells (NT) (*n* = 12; Mann–Whitney test). **(B)** Cell viability assessed using the CellTiter-Glo® luminescent assay, reported as ATP-dependent luminescence (Relative luminescence unit, RLU) after 24 h of treatment (*n* ≥ 10; Mann–Whitney test). **(C)** Representative flow cytometry histograms of mitochondrial reactive oxygen species (ROS) production. Quantification of mitochondrial ROS levels expressed as mean percentage fluorescence intensity (*n* = 3; unpaired *t*-test). **(D)** Propidium iodide (PI) staining cell cycle analyses. Results are expressed as mean percentage of cells in each cell cycle phase (*n* = 6; Mann–Whitney test). **(E)** Summary graph of wound closure rates in treated versus control cells; representative images from wound healing assay after 24 h of treatment (magnification: 4x; scale bar = 100 μm) and corresponding quantification of % area closure (*n* = 14; Mann–Whitney test). **(F)** Representative images of transwell migration assay after 24 h of treatment (magnification: 10×; scale bar = 50 μm) and quantification of migrated cells per field (*n* ≥ 5; Mann–Whitney test). Data are shown as mean ± SEM. *p < 0.05; **p < 0.01; ***p < 0.001.

MTT assay allowed us to evaluate the impact of *S. pratensis* on cell viability in terms of mitochondrial dehydrogenase activity and confirm that the extract exhibited cytotoxic effects on MDA-MB-231 cells. After 24 h of treatment, the number of viable cells decreased by 19% (*p* = 0.043, Figure 2A), and a treatment induced a 44% reduction in MDA-MB-231 (*p* = 0.0007, Supplementary Figure S1A). No significant effects were observed in MCF 10A viability at either time point (Figure 2A and Supplementary Figure S1A). To further validate *S. pratensis* cytotoxicity, we measured the adenosine triphosphate (ATP) production in viable MDA-MB-231 cells under stimulations. Coherently, treatment with *S. pratensis* for 24 h resulted in a 25% significant reduction of the ATP-associated luminescence in respect to control cells treated with dimethyl sulfoxide (DMSO) (*p* = 0.0002, Figure 2B). Furthermore, we evaluated whether the decrease in metabolic activity was associated with oxidative stress. Importantly, *S. pratensis* treatment induced an approximately 22-fold increase in mitochondrial ROS levels compared with vehicle-treated cells (*p* = 0.0003; Figure 2C). Cell cycle analysis was conducted to validate the antiproliferative effects of *S. pratensis* on MDA-MB-231 cells and revealed a significant accumulation of cells in the G0/G1 phase compared to the DMSO-treated control (*p* = 0.0087). This shift was accompanied by a corresponding decrease in the S population (*p* = 0.045, Figure 2D).

### 3.4 *Salvia pratensis* reduced motility in MDA-MB-231

The effects of *S. pratensis* on the proliferative and migratory properties of MDA-MB-231 cells were evaluated by wound-healing and Boyden chamber assay, respectively. In the wound-healing assay, gap closure was monitored over time (at the time of the treatment, and at the following 6, 24, and 30 h after *S. pratensis* stimulation). Experimental evidence demonstrated that treatment with *S. pratensis* significantly impaired wound closure, resulting in a 32% reduction in migration potential at 24 h (*p* = 0.0092, Figure 2E) and 21% at 30 h (*p* = 0.0282, Supplementary Figure S1B) compared to controls. To more specifically quantify their motility capacity, we employed a Boyden chamber system. After 24 h of treatment with *S. pratensis*, the number of migrated cells was significantly reduced by 39% (*p* = 0.0031) compared to controls (Figure 2F).

### 3.5 *Salvia pratensis* affected stemness, EMT, inflammation, oxidative stress and apoptosis

**Figure 3:**
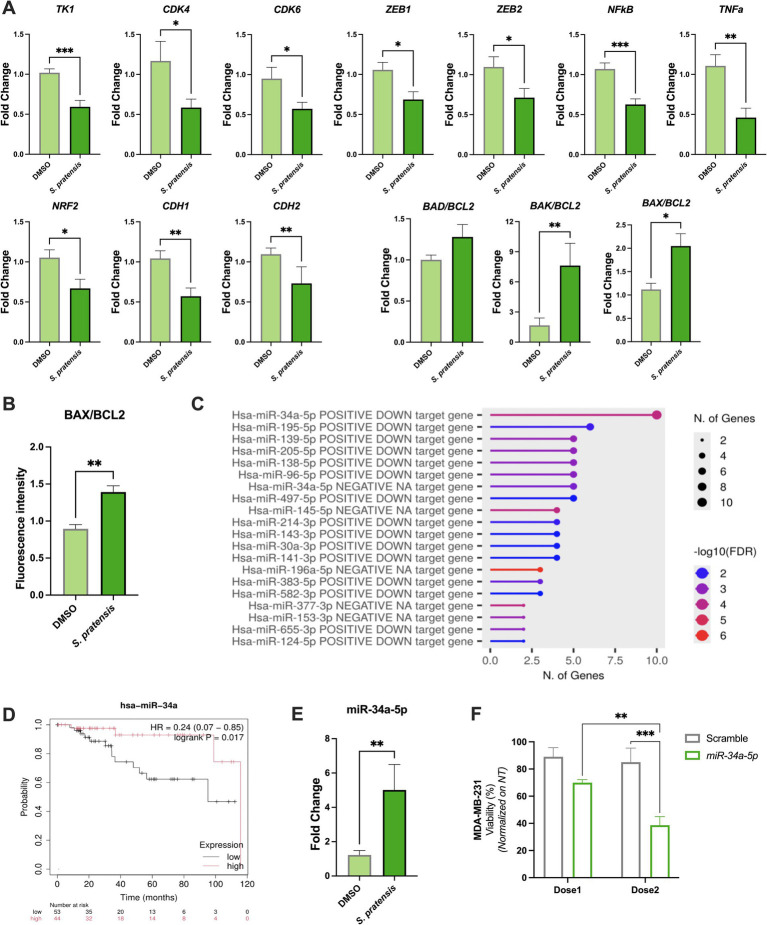
Impact of *S. pratensis* on MDA-MB-231 gene expression, protein modulation, and miRNA analysis. **(A)** Bar graphs showing the differential gene expression after 24 h of treatment reported as fold change compared to DMSO (*n* = 10; Mann–Whitney test). **(B)** BAX and BCL2 protein expression were quantified by confocal immunofluorescence and represented as fluorescence intensity ratio (*n* = 5; Mann–Whitney test). **(C)** Enrichment plot of the differentially expressed genes using Diana-TarBase ontology. In the plot, the most enriched miRNAs are reported sorted by the number of genes and colored by FDR value. **(D)** Kaplan–Meier plot of hsa-miR-34a. The data used are related to MDA-MB-231 cell line; two probability curves are reported, one associated with low relative expression of hsa-miR-34a (black), the other to high expression level (red). **(E)** hsa-miR-34a-5p relative expression levels upon the treatment with *S. pratensis.* **(F)** Cell viability of MDA-MB-231 cells transfected with miR-34a-5p or scramble control at Dose1 (0.6 μg/mL) and Dose2 (0.8 μg/mL), expressed as percentage of viability relative to non-transfected cells (*n* = 4; two-way ANOVA). Data are presented as mean ± SEM. *p < 0.05; **p < 0.01; ***p < 0.001.

To investigate the molecular mechanisms affected by *S. pratensis* treatment on MDA-MB-231, we quantified the expression of key genes involved in the pathways observed functionally impaired. Notably, treatment with *S. pratensis* led to significant downregulation of the proliferation markers *TK1* (*p* = 0.001), *CDK4* (*p* = 0.0338), and *CDK6* (*p* = 0.0218). Additionally, *S. pratensis* treatment induced a downregulation in stemness marker *ZEB1* (*p* = 0.0175) and *ZEB2* (*p* = 0.0195), inflammatory/oxidative stress-related factors *NFκB* (*p* = 0.0004), *TNFα* (*p* = 0.0028), *NRF2* (*p* = 0.0232), and epithelial–mesenchymal transition (EMT) markers *CDH1* (*p* = 0.0055) and *CDH2* (*p* = 0.0027). Apoptotic regulation was assessed by measuring *BAD*, *BAK, BAX* and *BCL2* genes expression levels. The *BAD/BCL2* ratio was increased by 28% compared to the control conditions. Importantly, *BAK/BCL2* and *BAX/BCL2* ratios were significantly upregulated after *S. pratensis* stimulation (*p* = 0.0082 and *p* = 0.0175, respectively) (Figure 3A). The pro-apoptotic effect of *S. pratensis* treatment was further confirmed by confocal immunofluorescence analysis, which showed a significant increase of BAX (*p* = 0.0317) associated with a downregulation of BCL2 protein expression (*p* = 0.0317) (Supplementary Figure S1D). Importantly, the BAX/BCL2 protein ratio resulted in an increase compared to the control (*p* = 0.0079, Figure 3B).

### 3.6 *Salvia pratensis* up-regulated miR-34a-5p expression in MDA-MB-231

The analyzed genes panel included several targets involved in key oncogenic processes, such as oxidative stress response, inflammatory signaling, cell proliferation, and EMT, which are central to TNBC pathophysiology (24). To further investigate the molecular key regulator underlying *S. pratensis* effects, we performed a miRNA over-representation analysis using the DIANA-TarBase ontology (15) on the described differentially expressed genes (*BAD*, *BCL2*, *CDH1*, *CDH2*, *CDK4*, *CDK6*, *TK1*, *NFkB*, *TNFα*, *ZEB1*, *ZEB2*). Among all enriched miRNAs, miR-34a-5p has 10 associations, resulting in the one with the highest number of significant associations (Figure 3C). This finding was corroborated by using miRTarBase ontology (16), as reported in Supplementary Figure S2B, where miR-34a-5p again emerged as the one with the highest number of associations. Note that the general lower number of miRNA-gene associations observed using miRTarBase (5 vs. 10) is due to the total number of associations curated in this last ontology.

Focusing on this specific miRNA, Kaplan–Meier survival analysis was performed in the TNBC subgroup (*n* = 97; selected from the total cohort of 1,078 patients in the TCGA cohort), shown in Figure 3D, highlighted that the down-regulated expression of miR-34a-5p was strongly related to reduced overall survival (*p* = 0.017). Moreover, analysis of the Database of Differentially Expressed miRNAs in Human Cancers (dbDEMC) (25) indicates that miR-34a-5p is the third most frequently reported miRNAs associated with breast cancer, after miR-21 and miR-155 (Supplementary Figures S2A,B). However, although miR-21 and miR-155 are also frequently reported, Kaplan–Meier survival analyses in TNBC revealed no significant correlation between their expression and patient survival (Supplementary Figure S2C), and no significant association between the analyzed genes.

Based on these results, we quantified miR-34a-5p expression in MDA-MB-231 cell line in response to *S. pratensis* treatment, obtaining a significant 4-fold upregulation of the miRNA compared to the controls (*p* = 0.0022) (Figure 3E). To further investigate the role of miR-34a-5p in *S. pratensis*-induced cell death, we performed gain-of-function experiments by transfecting MDA-MB-231 cells with miR-34a-5p mimic at two different concentrations (0.6 and 0.8 μg/mL) for 24 h. Importantly, the scramble control did not significantly affect cell viability, confirming the specificity of the experimental approach. In contrast, transfection with miR-34a-5p mimic reduced cell viability by 21.47% (*p* = 0.0652) compared to the corresponding scramble control. This effect was dose-dependent, with a more pronounced reduction observed at 0.8 μg/mL, where viability significantly decreased by 54.62% (*p* = 0.0005). Moreover, cells treated with 0.8 μg/mL exhibited significantly lower viability compared to those treated with 0.6 μg/mL (*p* = 0.0044), further supporting a dose-responsive effect of miR-34a-5p overexpression (Figure 3F).

## 4 Discussion

TNBC stands out as a particularly aggressive form of cancer affecting women around the world, characterized by a high rate of relapse and drug resistance. Recently, natural extracts have garnered attention as promising sources of bioactive compounds with potential anticancer properties (26). In this study, we investigated three plant species from the Italian flora (*Petasites paradoxus*, *Typha laxmannii*, and *Salvia pratensis*) as sources of bioactive molecules, traditionally used in edible preparations, herbal infusions, or functional plant-based products, with potential activity against TNBC. Among these, *S. pratensis* emerged as the most promising species, exhibiting a phytochemical composition and functional effects aligned with selective anticancer activity. While the precise contributions of individual phytochemicals remain to be fully elucidated, the present study provides important insights into the selective anticancer effects of these natural extracts and their potential biomedical relevance.

Untargeted metabolomics showed species-specific metabolic profiles reflecting different biosynthetic strategies. *P. paradoxus* and *T. laxmannii* were enriched in hydroxycinnamic acid derivatives, flavanols, and structural or defensive secondary metabolites, such as pyrrolizidine alkaloids and condensed tannins. Instead, *S. pratensis* appeared dominated by rosmarinic acid, salvianolic acids, and luteolin glycosides. These phenolic compounds are well-known for their specific antiproliferative, cytostatic, and cytotoxic activities in cancer models (27–29). Notably, the presence of sagerinic acid and other oxidative coupling products in *S. pratensis* suggests the activation of specialized biosynthetic pathways not detected in the other two species, further underscoring its distinct metabolic profile and potentially enhanced therapeutic properties. Indeed, this chemical specificity was reflected in the functional assays, in which only *S. pratensis* after 24 h of treatment selectively impaired cell viability in MDA-MB-231, without affecting the viability of MCF 10A cells. The lack of a significant effect at 48 h could indicate that the extract activity is exerted primarily within the first 24 h, and that longer-term effects could require repeated administration. In contrast, both *P. paradoxus* and *T. laxmannii* significantly reduced MCF 10A cell counts after 24 h, indicating a lack of selectivity in their cytotoxic effects.

Focusing on *S. pratensis*, we demonstrated that treatment significantly reduced metabolic activity in MDA-MB-231 cells, accompanied by elevated mitochondrial ROS production. Mitochondrial dysfunction and oxidative stress are established mechanisms of action for various anticancer therapies. Interestingly, *Salvia officinalis*, a species from the same order (*Lamiaceae*), has also been reported to exert ROS-mediated antiproliferative effects in cancer. However, our data demonstrate that *S. pratensis* specifically induces mitochondrial ROS accumulation, suggesting this cellular component as a key mediator of its cytotoxic action.

Elevated oxidative stress is known to interfere with cell cycle dynamics, which revealed G0/G1 arrest under *S. pratensis* stimulation in concomitance with a decrease in the proportion of cells in S phase. Mechanistically, these effects were sustained by the downregulation of key regulators of cell cycle progression, including *CDK4*, *CDK6*, and thymidine kinase 1 (*TK1*). Significantly, *S. pratensis* stimulated MDA-MB-231 downregulated *TK1* expressions, compared to the control. *TK1* overexpression has been associated with increased invasiveness and migratory capacity in breast cancer (30), suggesting that its downregulation may contribute to the impaired motility observed in wound healing and Boyden chamber assays. This was further supported by the observed proliferation and migration inhibition by *S. pratensis*. Furthermore, stemness-associated transcription factors *ZEB1* and *ZEB2* were significantly downregulated following treatment, suggesting a broader effect of *S. pratensis* on tumorigenic potential. Interestingly, *CDH1* expression was also reduced. While *CDH1* downregulation is traditionally associated with EMT, in this context, it occurs alongside the suppression of other aggressive EMT markers. This pattern could suggest that the extract induced an overall reduction in cellular aggressiveness and fitness, rather than selectively promoting EMT. Moreover, our findings underline the pro-apoptotic potential of *S. pratensis*, as demonstrated by the upregulation of the pro-apoptotic *BAX/BCL2* ratio at both the gene and protein levels. This effect was further supported by increased *BAD/BCL2* and *BAK/BCL2* ratios in treated cells compared to controls, collectively indicating a significant shift in the pro-apoptotic balance. While a direct mechanistic link between *TK1* suppression and apoptosis remains to be fully elucidated, previous studies suggest that *TK1* downregulation may indirectly reduce *BCL2* expression and enhance apoptotic responses.

A key finding of our study was the upregulation of the tumor-suppressive miR-34a-5p upon *S. pratensis* treatment. This modulation suggests a potential mechanism by which the extract exerts its selective anticancer activity, not only through direct cytotoxicity but also by influencing key regulatory networks. Consistently with our results, miR-34a-5p had already been associated with regulator genes associated with cell cycle arrest, apoptosis, and EMT, and its tumor-suppressive function in cancer. Its relevance in TNBC is further highlighted by the significant correlation between low miR-34a-5p expression and poorer patient survival. The upregulation of miR-34a-5p in MDA-MB-231 cells following *S. pratensis* treatment aligns with its reported anticancer role in breast cancer and reinforces its potential as a therapeutic mediator. Furthermore, gain-of-function experiments showed that overexpression of miR-34a-5p significantly reduced TNBC cell viability in a dose-dependent manner, providing functional evidence of its role in the cytotoxic effects induced by *S. pratensis*.

Finally, our study identifies *S. pratensis* as a promising source of plant-derived bioactive compounds able to attenuate *in vitro* TNBC cell aggressiveness through miR-34a-5p. Its common and traditional consumption in Mediterranean cuisine and herbal infusions supports a generally favorable safety profile compared with conventional chemotherapeutic agents, which are often burdened by significant adverse effects. Additional studies could better clarify bioavailability, pharmacokinetics, safety, and *in vivo* efficacy, providing a deeper understanding of its potential nutritional relevance and clinical implications in TNBC patients.

Overall, these results provide a proof-of-concept framework supporting future exploration of *S. pratensis* as a bioactive-rich nutraceutical candidate that may complement conventional therapeutic strategies in TNBC.

## Data Availability

The original contributions presented in the study are included in the article and supplementary material, further inquiries can be directed to the corresponding author.

## Footnotes

1
www.nbfc.it

## Glossary

TNBC: Triple-Negative Breast Cancer
NBFC: National Biodiversity Future Center
ROS: Reactive oxygen species
EMT: Epithelial-mesenchymal transition
ER: Estrogen receptor
PR: Progesterone receptor
HER2: Human epidermal growth factor receptor 2
UPLC-ESI-HRMS: Ultra-Performance Liquid Chromatography Electrospray Ionization High-Resolution Mass Spectrometry
LC–MS: Liquid Chromatography-Mass Spectrometry
BPC: Base peak chromatograms
*P. paradoxus*: *Petasites paradoxus*
*S. pratensis*: *Salvia pratensis*
*T. laxmannii*: *Typha laxmannii*
MTT: Methylthiazol tetrazolium
ATP: Adenosine triphosphate
DMSO: Dimethyl sulfoxide
*TK1*: *Thymidine kinase 1*
*CDK4*: *Cyclin dependent kinase 4*
*CDK6*: *Cyclin dependent kinase 6*
*BAD*: *BCL2 associated agonist of cell death*
*BAK*: *BCL2 Antagonist/Killer 1*
*BAX*: *BCL2 Associated X*
*ZEB1*: *Zinc finger E-box binding homeobox 1*
*ZEB2*: *Zinc finger E-box binding homeobox 2*
*NFkB*: *Nuclear factor kappa B subunit 1*
*TNFa*: *Tumor necrosis factor alpha*
*NFR2*: *Nuclear factor erythroid 2–related factor 2*
dbDEMC: Database of Differentially Expressed miRNAs in Human Cancers
FAST-DDA: Fast Data-Dependent Acquisition
qTOF: Quadrupole Time Of Flight
PDA: Photo Diode Array
DMEM: Dulbecco’s Modified Eagle Medium
FBS: Fetal bovine serum
hEGF: Human Epidermal Growth Factor
HEPES: 4-(2-hydroxyethyl)-1-piperazineethanesulfonic acid
PI: Propidium Iodide
RT-qPCR: Real-Time Quantitative PCR
FDR: False discovery rate
GEO: Gene Expression Omnibus
EGA: European Genome-phenome Archive
TCGA: The Cancer Genome Atlas
NT: Untreated
