# Supplementary material for: Salvia pratensis exhibits in vitro anti-cancer effects in triple-negative breast cancer through miR-34a-5p signaling
Source: Front Nutr. 2026 Mar 18;13:1786148. doi: 10.3389/fnut.2026.1786148 (PMC13038989; doi:10.3389/fnut.2026.1786148)
Supplement: Supplementary file 1 [file Data_Sheet_1.pdf]

Supplementary Material

1. Supplementary Methods

1.1. Plant material and extract preparation

Plants of *Petasites paradoxus* (Retz.) Baumg., *Salvia pratensis* L., and *Typha laxmannii* Lepech. were purchased from the institutional nursery "Centre for biodiversity and outside-forest activities" of Veneto Agricoltura (Montecchio Precalcino, Vicenza, Italy). For each species, leaves were sampled from two plants in the vegetative growth phase and pooled. The fresh material was immediately frozen in liquid nitrogen and ground using an A11 basic analytical mill (IKA-Werke, Staufen, Germany). About 1 g of frozen powder was extracted with 10 mL of LC-MS grade methanol (Honeywell, Seelze, Germany). The samples were vortexed for 30 s, sonicated on ice for 10 minutes in a 40-kHz ultrasonic bath (Soltec, Milano, Italy), and centrifuged at 14,000 x g for 10 minutes at 4°C. The recovered supernatants were split into 1 mL aliquots, each corresponding to 100 mg of fresh weight. One aliquot per species was analyzed through UPLC-ESI-HRMS for phytochemical profiling while the remaining aliquots were dried with a speed-vac system (Heto-Holten, Frederiksborg, Denmark). The dried extracts were re-solubilized in 200 µL of dimethyl sulfoxide (DMSO), for subsequent cell-based assays.

1.2. UPLC-ESI-HRMS analysis

The methanolic leaf extracts of the three species were diluted 1:50 (*S. pratensis* and *T. laxmannii*) or 1:10 (*P. paradoxus*) with LC-MS grade water (Honeywell) and passed through 0.22 µm Minisart filters (Sartorius-Stedim Biotech, Göttingen, Germany). Samples were analyzed in negative and positive ionization modes, by injecting 5 µL and 1-2 µL, respectively, in an Acquity I-Class UPLC connected to an eLambda 800 nm PDA detector and a Xevo G2-XS qTOF mass spectrometer equipped with ESI source (Waters). Separation in reverse phase conditions (C18), detection (in continuum mode) and fragmentation (with FAST-DDA analysis) of plant metabolites were performed with the methods previously described (1). Putative identification of plant metabolites was performed manually, by considering their accurate mass (deduced from the *m/z* ratio and isotopic pattern), retention time, and fragmentation pattern (from the FAST-DDA analysis). For each metabolite, the three orthogonal parameters were compared with a proprietary library of authentic standard compounds, with an in silico proprietary library of plant compounds or with scientific literature and public databases (*e.g.*, MassBank, https://massbank.eu/MassBank/Search; MoNA, https://mona.fiehnlab.ucdavis.edu/). When no information was available, metabolites were tentatively identified according to diagnostic fragments and neutral losses frequently observed for plant metabolites. The analysis in positive ionization mode was used to confirm the molecular ions detected in negative ionization mode and to detect and identify the pyrrolizdine alkaloids of *P. paradoxus*.

1.3. Cell lines culture and treatments

MDA-MB-231, a TNBC epithelial cell line (ICLC-Biologic Bank and Cell Factory, Italy, passage 35-45), and MCF 10A (ATCC, passage 20-30), a human non-tumorigenic epithelial breast cell line (2), were cultured in a humidified atmosphere with 5% CO2 and 37°C. MDA-MB-231 were maintained in Advanced Dulbecco's Modified Eagle Medium (DMEM) (Gibco, Life Technologies Carlsbad, California, USA) with 10% fetal bovine serum (FBS) (Microgem, London, United Kingdom), 1% Antibiotic-Antimycotic (100X) (Sigma-Aldrich, Missouri, USA) and 2 mM L-Glutamine (EuroClone, Milan, Italy). MCF 10A (ATCC) were cultured in Advanced DMEM supplemented with 10% FBS, 1% Antibiotic-Antimycotic (100X), 10 g/mL insulin (Sigma-Aldrich), 20 ng/mL human Epidermal Growth Factor (hEGF) (Sigma-Aldrich), 0.5 g/mL hydrocortisol (Sigma-Aldrich), 20 mM HEPES (4-(2-hydroxyethyl)-1-piperazineethanesulfonic acid) (EuroClone) and 2 mM L-glutamine (EuroClone). When the culture reached approximately 70-80% confluence, cells were stimulated for 24 and 48 hours with the resuspended extracts of *P. paradoxus*, *S. pratensis*, and *T. laxmannii* at the final concentration of 5 μg/mL, or DMSO (ITW Reagents, Monza, Italy).

1.4. Viability and proliferation assays

A total of $5x10^4$ cells per well were seeded on a 24-well plate and stimulated with natural extracts. After 24 and 48 hours, the cells were detached using 0.25% v/v Trypsin-EDTA (EuroClone) and counted with Trypan-blue exclusion method. Additionally, 10^4 cells/wells were seeded on a 96-well plate and treated to evaluate cellular viability performing the Methylthiazol tetrazolium (MTT) assay and the CellTiter-Glo® assay (Promega, G9681).

For MTT assay, MDA-MB-231 and MCF 10A were incubated with 10 μL of 50 mg/mL of MTT solution for 4 hours at 37°C. Then, the supernatant was removed and 100 μL/well of DMSO was added. The colorimetric reaction was read by GloMax® Discover Microplate Reader (Promega, Madison, WI, USA) at a wavelength of 540 nm. In CellTiter-Glo® assay, an equal volume of reaction reagent and cell culture medium was added to each well. The contents were mixed vigorously according to the manufacturer's instructions. The plate was incubated at room temperature for an additional 10 minutes and read at 490 nm by GloMax® Discover Microplate Reader.

1.5. Wound Healing Assay

MDA-MB-231 cells were seeded in 24-well plates (10^5 cells/wells). The day after seeding, a linear wound was created at the center of each well using a sterile pipette tip. The medium was then gently replaced with fresh complete medium supplemented with *S. pratensis* extract or DMSO. Images of each well were acquired at 0, 6, 24, and 30 hours after treatment using a phase-contrast microscope (10x and 20x objectives). The scratched area (A) was quantified by ImageJ Software. The migration of cells toward the wounds was expressed as a percentage of wound closure and calculated as follows: % of wound closure = $[(A_{t=0h}-A_t)/A_{t=0h}]\times 100$.

1.6. Migration assays in Boyden chamber

A total of $5x10^4$ cells were seeded into the upper compartment of a Boyden chamber in medium with 2% FBS (EuroClone, Italy). The lower chamber was filled with a complete growth medium to serve as a chemoattractant. Control conditions consisted of identical mediums (medium containing 2% or 10% FBS) in both the upper and lower chambers to assess baseline migration. After 24 hours of incubation at 37°C, non-migrated cells on the upper surface of the membrane were gently removed using a cotton swab. The migrated cells on the lower membrane surface were stained with 2% crystal violet (Bio-Optica, Milan, Italy) for 20 minutes. Three representative fields per well were imaged, and the number of migrated cells was quantified using ImageJ Software.

1.7. Cell cycle analysis

To evaluate cell cycle, 10^6 harvested MDA-MB-231 were fixed with 70% cold ethanol and incubated 30 minutes at 4°C. Cells were incubated with 5 µg/mL propidium iodide (Sigma Aldrich) in PBS (Sigma Aldrich) supplemented with 2 mM of EDTA, 2% FBS, 0.6% trypsin, and 0.6% NP40 (BDH, Dubai, U.A.E.) for 30 minutes at room temperature. Samples were acquired with FACS Celesta (BD Life Sciences, New Jersey, USA) and obtained data were analyzed using FlowJo Software v.10 (BD Life Sciences).

1.8. Mitochondrial ROS evaluation by MitoSOX® assay

ROS production was quantified using MitoSOX® Red Mitochondrial Superoxide Indicator assay (Invitrogen, Thermo Fisher Scientific, Waltham, MA, USA), following the manufacturer's instructions. 10^5 MDA-MB-231 cells were seeded per well in a 6-well plate and treated after 24 hours. The samples were analyzed using a FACS Celesta flow cytometer (BD Biosciences) and the obtained data were processed with FlowJo Software.

1.9. Gene expression analysis by Real-Time Quantitative PCR (RT-qPCR)

Total RNA was isolated from MDA-MB-231 cells using the TRIzol™ reagent (Life Technologies, Thermo Fisher Scientific) according to the manufacturer's protocol. RNA concentration and purity were assessed using a Micro-volume spectrometer (LifeReal, Zhejiang, China). For gene expression analysis, 500 ng of total RNA was reverse transcribed using SuperScript IV Reverse Transcriptase (Invitrogen, Thermo Fisher Scientific). The obtained cDNA was amplified using the CFX Connect Real-Time PCR Detection System (BioRad, Hercules, CA, USA) using SYBR™ Green Fast qPCR Mix (Fisher Molecular Biology, Rome, Italy), with gene-specific primers (**Table S2**). For miRNA quantification, 100 ng of total RNA was reverse transcribed using the Mir-X™ miRNA First Strand Synthesis Kit (TaKaRa, Diatech, Italy), miRNAs expression was then amplified using CFX Connect Real-Time PCR Detection System using SYBR™ Green Fast qPCR Mix, with specific primers (**Table S2**). Relative expression levels were calculated using the $2^{-\Delta\Delta Ct}$ method (3), normalized to *14S* gene, for the gene expression, and to *U6*, for miRNAs.

1.10 Immunofluorescence analysis

5x10^5 cells were seeded on sterile glass coverslips placed in 6-well plates, after 24 hours cells were treated with DMSO, or *S. pratensis* extract for an additional 24 hours. Cells were fixed in 4% paraformaldehyde (PFA, Sigma-Aldrich) for 10 minutes at room temperature and permeabilized with 0.3% TritonX-100/PBS (Sigma-Aldrich) for 30 minutes at room temperature. Samples were incubated for 1 hour at room temperature in a blocking solution of PBS containing 2% bovine serum albumin (BSA, Sigma-Aldrich) and 0.1% Tween-20 (SERVA, Heidelberg, Germany). Primary antibody (BAX sc-7480, lot #L0208; BCL2 sc-7382, lot #H1208; Santa Cruz Biotechnology, Dallas, TX, USA) incubation was performed overnight at 4°C in blocking solution. Cells were incubated for 1 hour at room temperature in the dark with DyLight 488-conjugated goat anti-mouse secondary antibody (A90-516D2, Bethyl Laboratories, Montgomery, TX, USA). Coverslips were mounted using DAPI-containing mounting medium (Thermo Fisher) and stored at 4°C. Imaging was performed using Stellaris confocal microscope (Leica). Images were quantified by Fiji software.

1.11 Over-representation analysis

The resulting list of differentially expressed genes was subjected to functional over-representation analysis using the ShinyGO tool (4).
Over-representation of gene ontology terms was evaluated using a hypergeometric distribution-based test, with multiple testing corrections applied via the Benjamini-Hochberg method. A Q-value threshold of 0.05 was set to control the false discovery rate (FDR) and minimize the likelihood of type I errors in the context of multiple comparisons. We set all the other parameters as the default value.
To investigate potential post-transcriptional regulation, enrichment of experimentally validated miRNA targets was assessed using two complementary resources: DIANA-TarBase (5) and miRTarBase (6). These databases provide high-confidence miRNA–mRNA interaction data derived from experimental assays such as reporter gene assays, RT-qPCR, and western blot. Particular attention was given to miRNAs with known tumor-suppressive roles relevant to cancer pathophysiology.

1.12 Kaplan-Meier Survival Analysis

Kaplan-Meier survival analysis was conducted to investigate the prognostic significance of miRNA expression profiles, using the Kaplan-Meier-plotter tool (7). Kaplan-Meier-plotter is a highly advanced online tool for survival analysis, performing all computations in real time rather than displaying pre-generated images. Gene expression profiles and survival data (both relapse-free and overall survival) are sourced from the Gene Expression Omnibus (GEO), the European Genome-phenome Archive (EGA), and The Cancer Genome Atlas (TCGA). Using this tool, the user can query different omics data types, including mRNA, miRNA, and others.
To assess the prognostic significance of a specific gene, patient samples are divided into two groups based on different expression level thresholds of the selected biomarker. These two groups are then compared using Kaplan-Meier survival curves. The analysis includes calculation of the hazard ratio with 95% confidence intervals and a log-rank p-value (7).

Starting from the 1078 original patients from TCGA, we restricted the analysis to the 97 subtypes of TNBC. For the Keplein-Meier plotter, we set all the other parameters as the default value. Note that since only three pre-selected miRNAs were analyzed, no correction for multiple comparisons was applied.

1.13 Statistical Data Analysis

Normal distribution was evaluated by the Shapiro–Wilk test before performing parametric or non-parametric tests in each experiment. The statistical analysis was performed using Mann-Whitney t-test or one-way ANOVA test. A $p<0.05$ was considered statistically significant. The statistical analysis was performed using Prism 7.

2. Supplementary Figures and Tables

2.1. Supplementary Figures

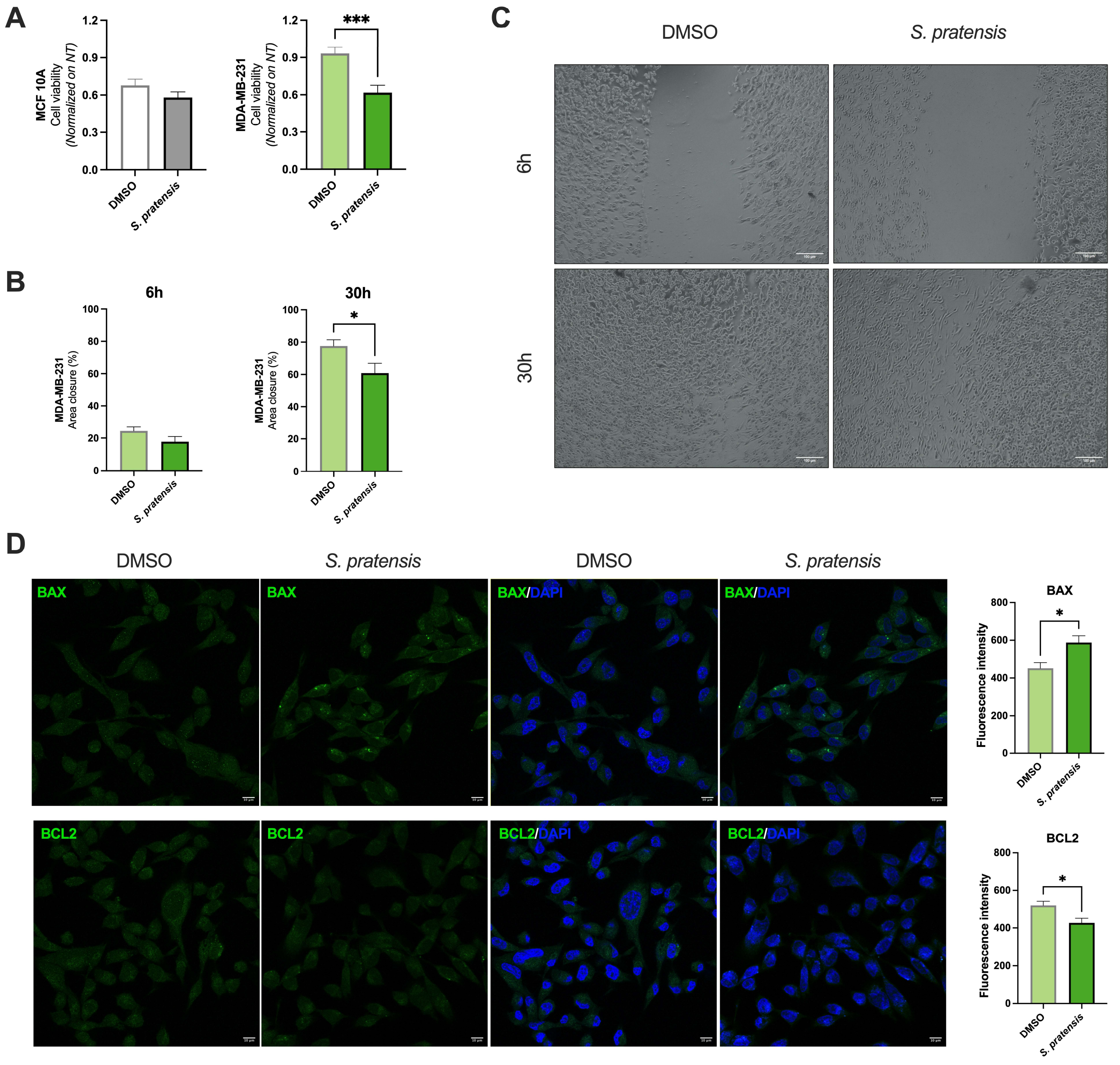

Fig.S1. Effects of *S. pratensis* extract on cell viability, migration, and apoptosis processes

(**A**) Cell viability measured by MTT assay, expressed as mean absorbance values after 48 hours of treatment (n=12; Mann–Whitney test). (**B**) Bar graph showing percentage wound closure over time, calculated as the reduction of cell-free area compared to baseline. (n=14; Mann–Whitney test). (**C**) Representative images from wound healing assay showing cell migration after 6 and 30 hours of treatment (magnification: 4x; scale bar=100 µm). (**D**) On the left: representative confocal images of BAX and BCL2 immunofluorescence detected with Alexa Fluor 488 (green) and merged with DAPI nuclear staining (blue) (magnification: 63x; scale bar=10 µm); on the right: quantification of BAX and BCL2 expression, reported as mean fluorescence intensity per cell (n=5; Mann–Whitney test). Data are shown as mean ± SEM. *p<0.05; ***p<0.001.

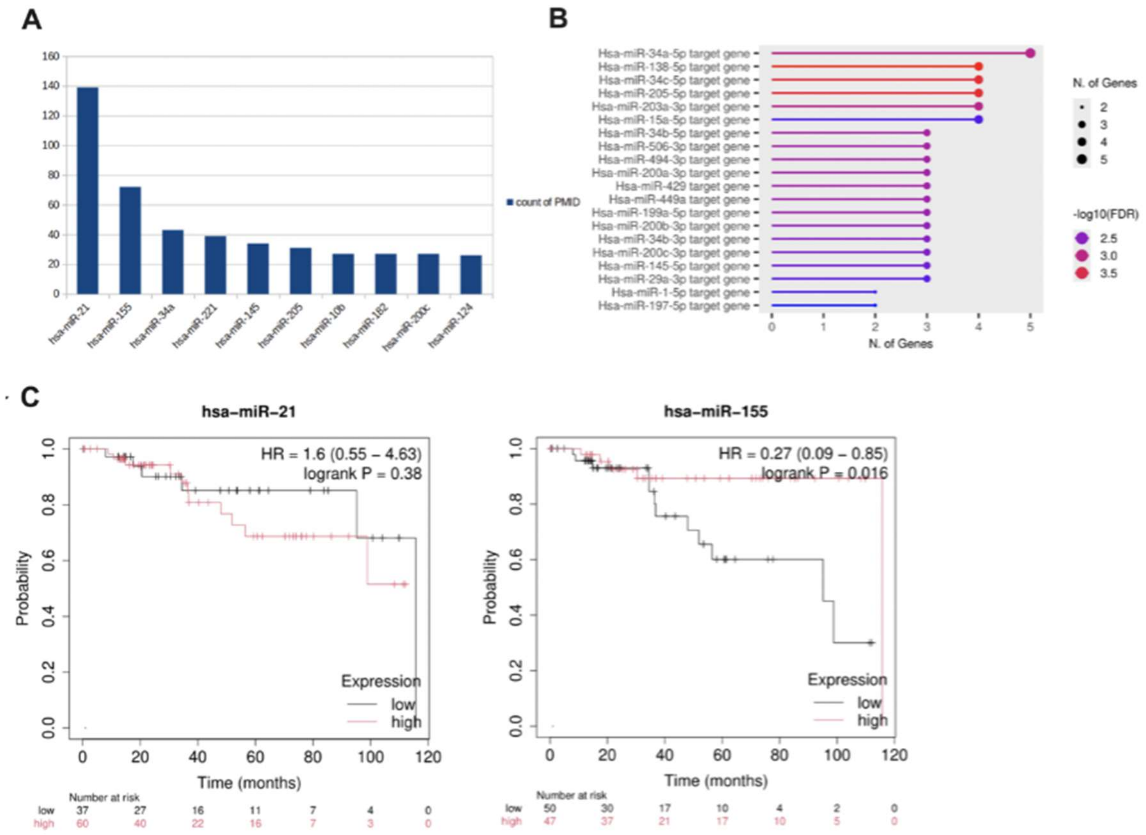

Fig.S2. Integrated analysis of breast cancer–associated miRNAs

(**A**) Bar-plot showing the total number of publications associated with the top 10 most studied miRNAs in breast cancer, ranked in descending order of publication count. Data were derived and re-elaborated from the dbMEC database, which curates experimentally supported associations between microRNAs and cancer types. (**B**) Enrichment plot of the differentially expressed genes using MirTarBase ontology. In the plot, the most enriched miRNAs are reported sorted by the number of genes and colored by FDR values. (**C**) Kaplan–Meier survival curves of breast cancer patients stratified into high (red) and low (black) miR-21 and hsa-miR-155 expression groups, using data from KMplot (www.kmplot.com). Only TNBC patients were selected, i.e. based on molecular similarity to the MDA-MB-231 cell line.

2.2 Supplementary Tables

***Table S1*. LC-MS features of the metabolites identified in *Petasites paradoxus*, *Salvia pratensis* and *Typha laxmannii* extracts.**

LC-MS features of the metabolites identified in *Petasites paradoxus, Salvia pratensis* and *Typha laxmannii* extracts. ID numbers refer to peaks indicated in Fig.1A-C; MSI level is reported in brackets next to the metabolite name (1, identification supported by authentic standard; 2, putative identification; 3, putative identification of the chemical class; 4, unidentified); Abbreviations: DP=degree of polymerization; n.d.=not detected. *= data are reported for negative ionization analysis, except for IDs 9,10,14,23,30 detected in positive ionization analysis; †=putative identification supported by diagnostic fragments (e.g., 96.959 m/z, HSO4-; 119.049 and 163.040 m/z, coumaric acid; 135.047 and 179.036 m/z, caffeic acid; 134.037 and 193.050 m/z, ferulic acid; 191.057 m/z, quinic acid), neutral losses (e.g., 79.957 Da, SO3; 42.01 Da, acetyl; 144.042 Da, hydroxymethylglutaryl; 132.042 Da, O-pentoside; 146.058 Da, O-deoxyhexoside; 162.053 Da, O-hexoside; 146.037 Da, coumaroyl; 162.032 Da, caffeoyl; 176.04 Da, feruloyl; 206.058 Da, sinapoyl) frequently observed in plant metabolites.

ID	Rt (min)	Putative identification[MSI]	Molecular class	Formula	UV-Vis, λ max (nm)	ESI molecular ion*	theoretical *m/z*	experimental *m/z*	Δppm	Confirming fragments	Reference for putative identification
1	0.92	Di-hexose [2]	Oligosaccharides	$C_{12}H_{22}O_{11}$	n.d.	$[M+HCOOH-H^+]^-$	387.1139	387.114	0.26	341.1084; 215.0318; 179.0578; 161.0464; 119.0344; 101.0243; 89.0222	MassBank (accession MSBNK-RIKEN-PR100500)
2	1.15	Oligosaccharide DP 3[2]	Oligosaccharides	$C_{18}H_{32}O_{16}$	n.d.	$[M+HCOOH-H^+]^-$	549.1667	549.167	0.55	503.160; 341.109	(8)
3	1.24	Oligosaccharide DP 4[2]	Oligosaccharides	$C_{24}H_{42}O_{21}$	n.d.	$[M+HCOOH-H^+]^-$	711.2195	711.22	0.70	665.213; 503.160; 485.1510; 341.109; 179.0572	(8)
4	1.97	Oligosaccharide DP 5[2]	Oligosaccharides	$C_{30}H_{52}O_{26}$	n.d.	$[M+HCOOH-H^+]^-$	873.2723	873.274	1.95	827.2667; 647.2021; 503.160; 485.1510; 341.109; 179.0572	(8)
5	2.40	Oligosaccharide DP 8[2]	Oligosaccharides	$C_{48}H_{82}O_{41}$	n.d.	$[M+HCOOH-H^+]^-$	1359.4308	1359.431	0.15	1313.4319; 1151.394; 989.324; 341.109; 179.057	†
6	2.57	Oligosaccharide DP 6[2]	Oligosaccharides	$C_{36}H_{62}O_{31}$	n.d.	$[M+HCOOH-H^+]^-$	1035.3251	1035.327	1.84	989.324; 899.2845; 665.213; 503.160; 341.109; 179.0572	†
7	4.10	5-O-Caffeoylquinic acid (Neochlorogenic acid)[1]	Hydroxycinnamic acid derivatives	$C_{16}H_{18}O_{9}$	242, 321	$[M-H^+]^-$	353.0873	353.087	-0.85	191.057; 179.0352; 177.020; 173.047; 161.027; 149.026; 135.046	

8	4.72	3-O-Caffeoylquinic acid (Chlorogenic acid)[(1)]	Hydroxycinnamic acid derivatives	$C_{16}H_{18}O_9$	248; 323	$[M-H^+]^-$	353.0873	353.087	-0.85	191.057; 179.0352; 177.020; 173.047; 161.027; 149.026; 135.046	
9	4.93	Petasitenine isomer[(2)]	Pyrrolizidine alkaloids	$C_{19}H_{27}NO_7$	n.d.	$[M+H^+]^+$	382.1866	382.1879	3.40	168.1019; 150.0905; 122.0607; 94.0652	(9)
10	5.57	Fukinotoxin (Petasitenine)[(2)]	Pyrrolizidine alkaloids	$C_{19}H_{27}NO_7$	n.d.	$[M+H^+]^+$	382.1866	382.1879	3.40	168.1019; 150.0905; 122.0607; 94.0652	(9)
11	5.71	Feruloylquinic acid[(2)]	Hydroxycinnamic acid derivatives	$C_{17}H_{20}O_9$	241, 323	$[M-H^+]^-$	367.1029	367.102	-2.45	191.054; 193. 051: 173.0449;134.0374	†
12	6.12	Rutin isomer[(2)]	Flavonols	$C_{27}H_{30}O_{16}$	253, 348	$[M-H^+]^-$	609.1456	609.146	0.66	300.026; 301.034; 271.0272; 255.0301	†
13	6.17	Quercetin-3-O-rutinoside (Rutin)[(1)]	Flavonols	$C_{27}H_{30}O_{16}$	254, 352	$[M-H^+]^-$	609.1456	609.146	0.66	300.026; 301.034; 271.0272; 255.0301	
14	6.23	Senkirkine[(1)]	Pyrrolizidine alkaloids	$C_{19}H_{27}NO_6$	n.d.	$[M+H^+]^+$	366.1917	366.1916	-0.27	168.1019; 150.0905; 122.0607; 94.0652	
15	6.32	Quercetin 3-O-galactoside (Hyperoside)[(1)]	Flavonols	$C_{21}H_{20}O_{12}$	254, 351	$[M-H^+]^-$	463.0876	463.087	-1.30	300.026; 301.034	
16	6.39	Quercetin 3-O-glucoside (Isoquercetin)[(1)]	Flavonols	$C_{21}H_{20}O_{12}$	254, 351	$[M-H^+]^-$	463.0876	463.087	-1.30	300.026; 301.034	

17	6.53	Fukinolic acid[2]	Hydroxycinnamic acid derivatives	$C_{20}H_{18}O_{11}$	248; 328	[M-H$^+$]$^-$	433.0771	433.077	-0.23	135.047; 179.036; 253.037; 271.047	(10)
18	6.66	Quercetin-O-(O-acetyl)-hexoside isomer[2]	Flavonols	$C_{23}H_{22}O_{13}$	253, 350	[M-H$^+$]$^-$	505.0982	505.098	-0.40	151.001; 255.030; 271.024; 300.030; 301.037; 463.087	†
19	6.75	Dicaffeoylquinic acid isomer[2]	Hydroxycinnamic acid derivatives	$C_{25}H_{24}O_{12}$	245, 323	[M-H$^+$]$^-$	515.118	515.1193	2.52	179.036; 191.057	†
20	6.90	Dicaffeoylquinic acid isomer[2]	Hydroxycinnamic acid derivatives	$C_{25}H_{24}O_{12}$	245, 323	[M-H$^+$]$^-$	515.118	515.119	1.94	179.036; 191.057	†
21	7.04	Quercetin-O-(O-acetyl)-hexoside isomer[2]	Flavonols	$C_{23}H_{22}O_{13}$	253; 350	[M-H$^+$]$^-$	505.098	505.098	0.00	179.034; 255.030; 271.024; 300.026; 301.034	†
22	7.17	Dicaffeoylquinic acid isomer[2]	Hydroxycinnamic acid derivatives	$C_{25}H_{24}O_{12}$	244, 325	[M-H$^+$]$^-$	515.1189	515.12	2.14	135.047; 179.036; 191.057	†
23	7.24	Acetylfukinotoxin (Neopetasitenine)[2]	Pyrrolizidine alkaloids	$C_{21}H_{29}NO_8$	n.d.	[M+H$^+$]$^+$	424.1971	424.198	2.12	382.187; 168.101; 150.0905; 122.0607	†
24	7.29	Quercetin-O-(O-caffeoyl)-hexoside[2]	Flavonols	$C_{30}H_{26}O_{15}$	251, 331	[M-H$^+$]$^-$	625.1193	625.12	1.12	255.030; 271.047;300.026; 301.034; 463.087	†
25	7.47	Dehydrofukinoside A[2]	Sesquiterpenes	$C_{21}H_{32}O_{10}S$	n.d.	[M-H$^+$]$^-$	475.1638	475.164	0.42	241.0043; 96.9598	†
26	7.67	Caffeoylferuloylquinic acid isomer[2]	Hydroxycinnamic acid derivatives	$C_{26}H_{26}O_{12}$	243, 325	[M-H$^+$]$^-$	529.1346	529.135	0.76	135.047; 179.036; 191.057; 193.053; 353.089; 367.105	†

27	7.73	Caffeoylferuloylquinic acid isomer(2)	Hydroxycinnamic acid derivatives	$C_{26}H_{26}O_{12}$	243, 325	$[M-H^+]^-$	529.1346	529.135	0.76	179.036; 191.057; 193.051	†
28	7.87	Caffeoylferuloylquinic acid isomer(2)	Hydroxycinnamic acid derivatives	$C_{26}H_{26}O_{12}$	245, 324	$[M-H^+]^-$	529.1346	529.1353	1.32	179.036; 191.057; 193.051	†
29	7.95	Caffeoylferuloylquinic acid isomer(2)	Hydroxycinnamic acid derivatives	$C_{26}H_{26}O_{12}$	246, 324	$[M-H^+]^-$	529.1346	529.1351	0.94	179.036; 191.057; 193.051	†
30	8.16	Acetylsenkirkine(2)	Pyrrolizidine alkaloids	$C_{21}H_{29}NO_7$	n.d.	$[M+H^+]^+$	408.2022	408.2007	-3.67	366.1878; 348.1787; 168.1019; 150.0905; 122.0607; 94.0652	†
31	8.81	Fukinoside A(2)	Sesquiterpenes	$C_{21}H_{34}O_{10}S$	n.d.	$[M-H^+]^-$	477.1794	477.179	-0.84	241.002; 96.9598	(11)
32	3.43	Danshensu (Salvianic acid A)(1)	Phenolic acids	$C_9H_{10}O_5$	235, 274	$[M-H^+]^-$	197.045	197.0458	4.06	179.0338; 151.0391; 135.0441; 134.0376; 123.0460; 122.0378; 107.0502	
33	4.14	Caffeoylthreonic acid isomer(2)	Hydroxycinnamic acid derivatives	$C_{13}H_{14}O_8$	244, 327	$[M-H^+]^-$	297.061	297.0614	1.35	179.036; 161.029; 135.030; 117.017; 75.006	(12)
34	4.24	Syringoyl hexose(2)	Phenolic acids	$C_{15}H_{20}O_{10}$	n.d.	$[M-H^+]^-$	359.0978	359.0982	1.11	299.0770; 239.0574; 211.0630; 197.0464: 182.0229; 153.0529	MassBank (accession MSBNK-RIKEN-PR309061)

35	4.31	Caffeoylthreonic acid isomer[(2)]	Hydroxycinnamic acid derivatives	$C_{13}H_{14}O_8$	244, 327	$[M-H^+]^-$	297.061	297.0614	1.35	179.036; 161.029; 135.030; 117.017; 75.006	(12)
36	4.39	Caffeoylthreonic acid isomer[(2)]	Hydroxycinnamic acid derivatives	$C_{13}H_{14}O_8$	244, 327	$[M-H^+]^-$	297.061	297.0614	1.35	179.036; 161.029; 135.030; 117.017; 75.006	(12)
37	4.79	Coumaroyl hexose[(2)]	Hydroxycinnamic acid derivatives	$C_{15}H_{18}O_8$	n.d.	$[M-H^+]^-$	325.0923	325.0927	1.23	163.0400; 119.0493	†
38	5.14	Caffeoylthreonic acid isomer[(2)]	Hydroxycinnamic acid derivatives	$C_{13}H_{14}O_8$	244, 327	$[M-H^+]^-$	297.061	297.0614	1.35	179.036; 161.029; 135.030; 117.017; 75.006	(12)
39	5.23	Benzyl alcohol-(O-pentosyl)-hexoside[(2)]	Benzyl alcohols	$C_{18}H_{26}O_{10}$	n.d.	$[M-H^+]^-$	447.1502	447.1505	0.67	401.1436; 269.1034; 161.0443	MassBank (accession MSBNK-RIKEN-PR309417)
40	5.27	Tuberonic acid-O-hexoside[(2)]	Jasmonates	$C_{18}H_{28}O_9$	n.d.	$[M-H^+]^-$	387.1655	387.1664	2.32	243.8787; 225.1094; 207.1033; 163.1131	(13)
41	5.37	Feruloylthreonic acid[(2)]	Hydroxycinnamic acid derivatives	$C_{14}H_{16}O_8$	245, 327	$[M-H^+]^-$	311.0767	311.0769	0.64	193.0505; 178.0274; 149.0610; 135.0299; 134.0376; 117.0192	(12)
42	5.42	Rosmarinic acid hydrate (Danshensuan C)[(2)]	Hydroxycinnamic acid derivatives	$C_{18}H_{18}O_9$	233, 279	$[2M-H^+]^-$	755.1823	755.1838	1.99	377.0887; 359.0787; 197.0464; 179.0338; 161.0235; 135.0441; 133.0301	(14)

43	5.81	Rosmarinic acid derivative isomer(3)	Hydroxycinnamic acid derivatives	$C_{27}H_{26}O_{14}$	n.d.	$[M-H^{+}]^{-}$	573.1244	573.1243	-0.17	511.1243; 377.0847; 359.0749; 331.0833; 271.0609; 197.0436; 179.0338; 161.0235; 137.0254; 135.0465	†
44	5.87	Rosmarinic acid derivative isomer(3)	Hydroxycinnamic acid derivatives	$C_{27}H_{26}O_{14}$	n.d.	$[M-H^{+}]^{-}$	573.1244	573.1243	-0.17	511.1243; 377.0847; 359.0749; 331.0833; 271.0609; 197.0436; 179.0338; 161.0235; 137.0254; 135.0465	†
45	6.21	Salvianolic acid A(2)	Hydroxycinnamic acid derivatives	$C_{26}H_{22}O_{10}$	274	$[M-H^{+}]^{-}$	493.1135	493.1153	3.65	313.0726; 295.0627; 203.0354; 197.0464; 185.0256; 159.0455; 135.0441; 109.0307	(15)
46	6.51	Luteolin-7-O-glucoside (Cynaroside)(1)	Flavones	$C_{21}H_{20}O_{11}$	255, 348	$[M-H^{+}]^{-}$	447.0927	447.0943	3.58	285.0384; 284.0338; 269.1403; 151.0039	
47	6.66	Rosmarinic acid-O-hexoside isomer(2)	Hydroxycinnamic acid derivatives	$C_{24}H_{26}O_{13}$	248, 291, 320	$[M-H^{+}]^{-}$	521.1295	521.1302	1.34	359.075; 341.086; 323.077; 197.046; 179.034; 161.023; 135.044	†

48	6.89	Rosmarinic acid-O-hexoside isomer[(2)]	Hydroxycinnamic acid derivatives	$C_{24}H_{26}O_{13}$	n.d.	$[M-H^+]^-$	521.1295	521.1302	1.34	359.075; 341.086; 323.077; 197.046; 179.034; 161.023; 135.044	†
49	6.99	Sagerinic acid[(2)]	Hydroxycinnamic acid derivatives	$C_{36}H_{32}O_{16}$	n.d.	$[M-H^+]^-$	719.1612	719.1615	0.42	161.026; 359.075; 197.044; 179.036; 341.067; 135.044	(16)
50	7.19	Luteolin-O-(acetyl)-hexoside[(2)]	Flavones	$C_{23}H_{22}O_{12}$	253, 347	$[M-H^+]^-$	489.1033	489.1029	-0.82	447.090; 285.042; 284.0338	†
51	7.34	Salvianolic acid K[(2)]	Hydroxycinnamic acid derivatives	$C_{27}H_{24}O_{13}$	243, 286, 323	$[M-H^+]^-$	555.1139	555.1139	0.00	493.115; 359.079; 295.059; 197.046; 179.034 ; 161.026; 135.046	(17)
52	7.41	Rosmarinic acid[(1)]	Hydroxycinnamic acid derivatives	$C_{18}H_{16}O_8$	234, 328	$[2M-H^+]^-$	719.1612	719.1615	0.42	359. 0767; 197.046; 179.036; 161.026; 135.046; 133.030; 123.046	
53	7.79	Lithospermic acid isomer[(2)]	Hydroxycinnamic acid derivatives	$C_{27}H_{22}O_{12}$	245, 324	$[M-H^+]^-$	537.1033	537.1034	0.19	493.1153; 359.0787; 295.0591; 197.0464; 179.0365; 161.0235; 135.0441	(17)
54	7.96	Chrysoeriol-(hydroxy-methylglutaryl)-O-hexoside[(2)]	Flavones	$C_{28}H_{30}O_{15}$	250, 342	$[M-H^+]^-$	605.1506	605.149	-2.64	503.119; 461.107; 299.056; 284.034; 255.030	†

55	8.17	Oct-3-en-yl-O-(O-pentosyl)-hexoside[(2)]	Fatty alcohols	$C_{19}H_{34}O_{10}$	n.d.	[M+HCOOH-H⁺]⁻	467.2128	467.2137	1.93	421.208; 289.169; 161.044; 149.046; 131.033; 113.024; 101.022	MassBank (accession MSBNK-BS-BS001011)
56	8.37	Methylrosmarinate[(2)]	Hydroxycinnamic acid derivatives	$C_{19}H_{18}O_8$	245, 327	$[M-H^+]^-$	373.0923	373.0932	2.41	197.046; 179.036; 175.039; 160.017; 135.044; 123.046	(12)
57	9.24	Salvianolic acid L[(2)]	Hydroxycinnamic acid derivatives	$C_{36}H_{30}O_{16}$	n.d.	$[M-H^+]^-$	717.1456	717.1465	1.25	519.095; 338.052; 179.034; 161.023; 135.044	(18)
58	3.39	B-type (epi)gallocatechin - (epi)catechin dimer[(2)]	Proanthocyanidins	$C_{30}H_{26}O_{13}$	n.d.	$[M-H^+]^-$	593.1295	593.1297	0.34	467.0987; 425.0894; 407.0804; 303.0507; 289.0712; 177.0185; 125.0222	(19)
59	3.52	Gallocatechin[(1)]	Flavan-3-ols	$C_{15}H_{14}O_7$	n.d.	$[M-H^+]^-$	305.0661	305.067	2.95	261.0795; 221.0469; 219.0674; 179.0365; 125.0245	
60	3.58	Caffeoyl hexose sulfate[(2)]	Hydroxycinnamic acid derivatives	$C_{15}H_{18}O_{12}S$	n.d.	$[M-H^+]^-$	421.0441	421.0439	-0.48	341.0860; 241.0043; 179.0338; 135.0441;	†
61	3.67	B-type (epi)gallocatechin - (epi)catechin dimer[(2)]	Proanthocyanidins	$C_{30}H_{26}O_{13}$	n.d.	$[M-H^+]^-$	593.1295	593.1297	0.34	467.0987; 425.0894; 407.0804; 303.0507; 289.0712; 177.0185; 125.0222	(19)

62	4.02	B-type (epi)gallocatechin-(epi)catechin dimer [(2)]	Proanthocyanidins	$C_{30}H_{26}O_{13}$	n.d.	$[M-H^+]^-$	593.1295	593.1297	0.34	467.0987; 425.0894; 407.0804; 303.0507; 289.0712; 177.0185; 125.0222	(19)
63	4.18	B-type (epi)catechin-(epi)catechin dimer[(2)]	Proanthocyanidins	$C_{30}H_{26}O_{12}$	n.d.	$[M-H^+]^-$	577.1346	577.1343	-0.52	451.1062; 425.0894; 407.0763; 289.0712; 125.0245	(20)
64	4.29	B-type (epi)catechin-(epi)catechin dimer[(2)]	Proanthocyanidins	$C_{30}H_{26}O_{12}$	236, 280	$[M-H^+]^-$	577.1346	577.1343	-0.52	451.1062; 425.0894; 407.0763; 289.0712; 125.0245	(20)
65	4.56	B-type (epi)catechin-(epi)catechin dimer[(2)]	Proanthocyanidins	$C_{30}H_{26}O_{12}$	n.d.	$[M-H^+]^-$	577.1346	577.1343	-0.52	451.1062; 425.0894; 407.0763; 289.0712; 125.0245	(20)
66	4.63	Quercetin-di-O-hexoside-O-deoxyhexoside[(2)]	Flavonols	$C_{33}H_{40}O_{21}$	n.d.	$[M+HCOOH-H^+]^-$	817.2039	817.2032	-0.86	771.2015; 609.1468; 462.0786; 301.0339	†
67	4.66	Catechin[(1)]	Flavan-3-ols	$C_{15}H_{14}O_6$	238, 280	$[M-H^+]^-$	289.0712	289.0712	0.00	245.0819; 205.0497; 203.0733; 125.0245; 123.0460; 109.0286	
68	4.69	Eriodictyol-di-C-hexoside[(2)]	Flavanones	$C_{27}H_{32}O_{16}$	n.d.	$[M-H^+]^-$	611.1612	611.1589	-3.76	521.1302; 491.1221; 401.0903; 371.0797;	(21)

										329.0866; 269.0664; 239.0574; 209.0459	
69	4.79	B-type (epi)afzelechin–(epi)catechin dimer[2]	Proanthocyanidins	$C_{30}H_{26}O_{11}$	240, 280	$[M-H^+]^-$	561.1397	561.1393	-0.71	435.1062; 289.0712; 245.0787; 125.0245	(20)
70	4.88	Caffeic acid sulfate[2]	Hydroxycinnamic acid derivatives	$C_9H_8O_7S$	n.d.	$[M-H^+]^-$	258.9912	258.9909	-1.16	215.0031; 179.0365; 135.0465; 96.9578	†
71	4.95	Caffeoylshikimic acid-O-hexoside[2]	Hydroxycinnamic acid derivatives	$C_{22}H_{26}O_{13}$	n.d.	$[M-H^+]^-$	497.1295	497.13	1.01	335.0763; 179.0365; 161.0235; 135.0465	†
72	5.00	Epicatechin-(4β □8)-epicatechin (Procyanidin B2)[1]	Proanthocyanidins	$C_{30}H_{26}O_{12}$	238, 277	$[M-H^+]^-$	577.1346	577.1343	-0.52	451.1062; 425.0894; 407.0763; 289.0712; 125.0245	
73	5.23	Apigenin-6,8-di-C-glucoside (Vicenin 2)[1]	Flavones	$C_{27}H_{30}O_{15}$	272, 330	$[M-H^+]^-$	593.1506	593.1497	-1.52	503.1187; 473.1071; 383.0777; 353.0657; 325.0706; 297.0770	
74	5.33	Epicatechin[1]	Flavan-3-ols	$C_{15}H_{14}O_6$	237, 278	$[M-H^+]^-$	289.0712	289.0712	0.00	245.0819; 205.0497; 203.0733; 125.0245; 123.0460; 109.0286	
75	5.42	B-type (epi)afzelechin–(epi)catechin dimer[2]	Proanthocyanidins	$C_{30}H_{26}O_{11}$	243, 280	$[M-H^+]^-$	561.1397	561.1393	-0.71	435.1062; 409.0939; 299.0557; 287.0564;	(20)

										273.0763; 161.0235 ; 125.0245	
76	5.46	Caffeoylshikimic acid[2]	Hydroxycinnamic acid derivatives	$C_{16}H_{16}O_8$	242, 325	$[M-H^+]^-$	335.0767	335.0772	1.49	179.0338; 161.0235; 135.0441	MoNA (accession Polyphenol000391)
77	5.50	Epicatechin-(4β □8)-epicatechin-(4β □8)-epicatechin (Procyanidin C1)[1]	Proanthocyanidins	$C_{45}H_{38}O_{18}$	243, 277	$[M-H^+]^-$	865.198	865.1997	1.96	739.1617; 577.1343; 407.0763; 287.0564	
78	5.58	B-type (epi)afzelechin–(epi)catechin dimer[2]	Proanthocyanidins	$C_{30}H_{26}O_{11}$	242, 277	$[M-H^+]^-$	561.1397	561.1393	-0.71	435.1062; 409.0939; 299.0557; 287.0564; 273.0763; 161.0235 ; 125.0245	(20)
79	5.81	B-type (epi)afzelechin-(epi)catechin-(epi)catechin trimer[2]	Proanthocyanidins	$C_{38}H_{42}O_{22}$	243, 280	$[M-H^+]^-$	849.2089	849.2068	-2.47	723.1627; 697.1588; 577.1343; 561.1442; 407.0763; 287.0564; 161.0235; 125.0245	(22)
80	5.95	(epi)afzelechin[2]	Flavan-3-ols	$C_{15}H_{14}O_5$	n.d.	$[M-H^+]^-$	273.0763	273.0763	0.00	255.0661; 229.0880; 205.0878; 189.0562; 187.0774; 97.0284	(23)

81	5.98	B-type (epi)afzelechin-(epi)afzelechin dimer[(2)]	Proanthocyanidins	$C_{30}H_{26}O_{10}$	243, 274	$[M-H^+]^-$	545.1448	545.1445	-0.55	419.1143; 409.0939; 312.0631; 273.0763; 125.0245	(22)
82	6.69	Kaempferol-3-O-rutinoside (Nictoflorin)[(1)]	Flavonols	$C_{27}H_{30}O_{15}$	264, 347	$[M-H^+]^-$	593.1506	593.1516	1.69	285.0418; 255.0301; 227.0358	
83	6.80	Isorhamnetin-3-O-rutinoside (Narcissoside)[(1)]	Flavonols	$C_{28}H_{32}O_{16}$	252, 355	$[M-H^+]^-$	623.1612	623.161	-0.32	315.0493; 300.0263; 299.0203; 271.0238; 255.0269	
84	7.30	Kaempferol-methylether-O-hexoside[(2)]	Flavonols	$C_{22}H_{22}O_{11}$	n.d.	$[M-H^+]^-$	461.1084	461.108	-0.87	446.0865; 298.0479; 283.0240; 255.0301	(24)
85	7.84	Unidentified with sinapoyl moiety[(4)]	-	–	n.d.	-	-	755.3131	#VALOR E!	223.0598; 205.0497; 190.0283; 175.0038; 164.0476; 149.0186	†
86	7.97	Unidentified with feruloyl moiety[(4)]	-	–	n.d.	-	-	725.2997	#VALOR E!	193.0505; 175.0389; 160.0148; 149.0585; 134.0376	†
87	10.06	Coumaroylferuloyl glycerol[(2)]	Hydroxycinnamic acid derivatives	$C_{22}H_{22}O_8$	n.d.	$[M-H^+]^-$	413.1236	413.1233	-0.73	249.0807; 193.0534; 177.0566; 145.0307; 134.0376; 119.0493	(25)

Table S2. List of primer sequences of genes and microRNA for qRT-PCR

Target	Forward	Reverse
14S	GGCAGACCGAGATGAATCCTCA	CAGGTCCAGGGGTCTTGGTCC
TK1	GGGGCAGATCCAGGTGATTC	GCATACTTGATCACCAGGCACTT
CDK4	ACGTCTGTGCCACATCCC	TCAGATCCTTGATCGTTTCGGC
CDK6	AACCTCAGTGGTCGTCACGCTGTG	GCTGGACTGGAGCAAGACTTCGGG
BAD	CCCAGAGTTTGAGCCGAGTG	CCCATCCCTTCGTCGTCCT
BCL2	GATTGTGGCCTTCTTTGAG	CAAACTGAGCAGAGTCTTC
BAX	CCTGTGCACCAAGGTGCCGGAACT	CCACCCTGGTCTTGGATCCAGCCC
BAK	CTTGGAGAGGCAAGAGACTG	CAGGGATGGGTGAGAGCCTT
ZEB1	GCACCTGAAGAGGACCAGAG	TGCATCTGGTGTTCCATTTT
ZEB2	CATGAACCCATTTAGTGCCA	AGCAAGTCTCCCTGAAATCC
NFkB	CAACAGCAGATGGCCCATACC	CGAAATCCTCTCTGTTTAGGTTGCTCTA
TNFa	AAGAGTTCCCCAGGGACCT	GGTTCAGCCACTGGAGCTG
NRF2	CAGCGACGGAAAGAGTATGA	TGGGCAACCTGGGAGTAG
CDH1	GCTGAGCTGGACAGGGAGGA	ATGGGGGCGTTGTCATTCAC
CDH2	CGAGCCGCCTGCGCTGCCAC	CGCTGCTCTCCGCTCCCCGC
hsa-miR-34a-5p	TGGCAGTGTCTTAGCTGGTTGT	
Scrambled miRNA	ATGATGTCCTTCTAGTACGCATC	

4 SI References

1. Giammona A, Commisso M, Bonanomi M, Remedia S, Avesani L, Porro D, Gaglio D, Bertoli G, Lo Dico A. A Novel Strategy for Glioblastoma Treatment by Natural Bioactive Molecules Showed a Highly Effective Anti-Cancer Potential. *Nutrients* (2024) 16:2389. doi: 10.3390/nu16152389
2. Bertoli G, Cava C, Diceglie C, Martelli C, Rizzo G, Piccotti Francesca and Ottobrini L, Castiglioni I. MicroRNA-567 dysregulation contributes to carcinogenesis of breast cancer, targeting tumor cell proliferation, and migration. *Breast Cancer Res Treat* (2017) 161:605–616.
3. Livak KJ, Schmittgen TD. Analysis of Relative Gene Expression Data Using Real-Time Quantitative PCR and the $2^{-\Delta\Delta CT}$ Method. *Methods* (2001) 25:402–408. doi: 10.1006/meth.2001.1262
4. Ge SX, Jung D, Yao R. ShinyGO: a graphical gene-set enrichment tool for animals and plants. *Bioinformatics* (2020) 36:2628–2629.
5. Skoufos G, Kakoulidis P, Tastsoglou Spyros and Zacharopoulou E, Kotsira V, Miliotis M, Mavromati G, Grigoriadis D, Zioga M, Velli A, Koutou I, et al. TarBase-v9.0 extends experimentally supported miRNA-gene interactions to cell-types and virally encoded miRNAs. *Nucleic Acids Res* (2024) 52:D304–D310.
6. Cui S, Yu S, Huang H-Y, Lin Y-C-D, Huang Y, Zhang B, Xiao J, Zuo H, Wang J, Li Z, et al. miRTarBase 2025: updates to the collection of experimentally validated microRNA-target interactions. *Nucleic Acids Res* (2025) 53:D147–D156.
7. Stalpers LJA, Kaplan EL. Edward L. kaplan and the Kaplan-Meier survival curve. *BSHM Bull* (2018) 33:109–135.
8. Song J, Liu Y, Yin X, Nan Y, Shi Y, Chen X, Liang H, Zhang J, Ma B. Isolation and structural elucidation of prebiotic oligosaccharides from Ziziphi Spinosae Semen. *Carbohydr Res* (2023) 534:108948. doi: 10.1016/j.carres.2023.108948
9. Kitajima M, Okabe K, Yoshida M, Nakabayashi R, Saito K, Kogure N, Takayama H. New otonecine-type pyrrolizidine alkaloid from Petasites japonicus. *J Nat Med* (2019) 73:602–607. doi: 10.1007/s11418-019-01285-9
10. Kim SM, Kang SW, Jeon J, Jung Y, Kim CY, Pan CH, Um B. Rapid identification and evaluation of antioxidant compounds from extracts of *Petasites japonicus* by hyphenated-HPLC techniques. *Biomedical Chromatography* (2012) 26:199–207. doi: 10.1002/bmc.1646
11. Yoshikawa M, Morikawa T, Tanaka J, Shimoda H. Medicinal Foodstuffs. XXXII. Novel Sesquiterpene Glycoside Sulfate, Fukinoside A, with Antiallergic Activity from Japanese Butterbur (Petasites japonicus). *Heterocycles* (2006) 68:2335. doi: 10.3987/COM-06-10858
12. Srećković N, Mišić D, Gašić U, Matić SLj, Katanić Stanković JS, Mihailović NR, Monti DM, D'Elia L, Mihailović V. Meadow sage (Salvia pratensis L.): A neglected sage species with valuable phenolic compounds and biological potential. *Ind Crops Prod* (2022) 189:115841. doi: 10.1016/j.indcrop.2022.115841
13. Rodríguez-Pérez C, Gómez-Caravaca AM, Guerra-Hernández E, Cerretani L, García-Villanova B, Verardo V. Comprehensive metabolite profiling of Solanum tuberosum L. (potato) leaves by HPLC-ESI-QTOF-MS. *Food Research International* (2018) 112:390–399. doi: 10.1016/j.foodres.2018.06.060
14. Cao J-L, Wang S-S, Hu H, He C-W, Wan J-B, Su H-X, Wang Y-T, Li P. Online comprehensive two-dimensional hydrophilic interaction chromatography × reversed-phase liquid chromatography coupled with hybrid linear ion trap Orbitrap mass spectrometry for the analysis of phenolic acids in Salvia miltiorrhiza. *J Chromatogr A* (2018) 1536:216–227. doi: 10.1016/j.chroma.2017.09.041
15. Li C, Yang J, Tong X, Zhao C, He Y, Wan H. Precursor ion scan enhanced rapid identification of the chemical constituents of Danhong injection by liquid chromatography–tandem mass spectrometry: An integrated strategy. *J Chromatogr A* (2019) 1602:378–385. doi: 10.1016/j.chroma.2019.04.023
16. Ribeiro A, Caleja C, Barros L, Santos-Buelga C, Barreiro MF, Ferreira ICFR. Rosemary extracts in functional foods: extraction, chemical characterization and incorporation of free and microencapsulated forms in cottage cheese. *Food Funct* (2016) 7:2185–2196. doi: 10.1039/C6FO00270F

17. Grzegorczyk-Karolak I, Krzemińska M, Kiss AK, Olszewska MA, Owczarek A. Phytochemical Profile and Antioxidant Activity of Aerial and Underground Parts of Salvia bulleyana Diels. Plants. *Metabolites* (2020) 10:497. doi: 10.3390/metabo10120497
18. Beltrán-Noboa A, Proaño-Ojeda J, Guevara M, Gallo B, Berrueta LA, Giampieri F, Perez-Castillo Y, Battino M, Álvarez-Suarez JM, Tejera E. Metabolomic profile and computational analysis for the identification of the potential anti-inflammatory mechanisms of action of the traditional medicinal plants Ocimum basilicum and Ocimum tenuiflorum. *Food and Chemical Toxicology* (2022) 164:113039. doi: 10.1016/j.fct.2022.113039
19. Escobar-Avello D, Lozano-Castellón J, Mardones C, Pérez AJ, Saéz V, Riquelme S, von Baer D, Vallverdú-Queralt A. Phenolic Profile of Grape Canes: Novel Compounds Identified by LC-ESI-LTQ-Orbitrap-MS. *Molecules* (2019) 24:3763. doi: 10.3390/molecules24203763
20. Prado DG, Justino AB, Silva T da C, de Morais SAL, Martins MM, Santos P de S, Cunha LCS, de Sousa RMF, de Aquino FJT, Espindola FS, et al. Phytochemical Profiling by HPLC-ESI-MS/MS and In Vitro Investigation of the Antidiabetic Activity of *Cassia bakeriana* Bark Extract and Fractions. *Journal of Mass Spectrometry* (2024) 59: doi: 10.1002/jms.5099
21. De Beer D, Schulze A, Joubert E, De Villiers A, Malherbe C, Stander M. Food Ingredient Extracts of Cyclopia subternata (Honeybush): Variation in Phenolic Composition and Antioxidant Capacity. *Molecules* (2012) 17:14602–14624. doi: 10.3390/molecules171214602
22. Hamed AI, Al-Ayed AS, Moldoch J, Piacente S, Oleszek W, Stochmal A. Profiles analysis of proanthocyanidins in the argun nut (*Medemia argun* —an ancient Egyptian palm) by LC–ESI–MS/MS. *Journal of Mass Spectrometry* (2014) 49:306–315. doi: 10.1002/jms.3344
23. Hernández M, Castañeta G, Simirgiotis MJ, Sepulveda B, Areche C. Comprehensive Phytochemical Profile of Leaves, Stems and Fruits from *Orthopterygium huaucui* (A. Gray) Hemsl. and their Antioxidant Activities. *Chem Biodivers* (2024) 21: doi: 10.1002/cbdv.202400746
24. Falcão SI, Vale N, Gomes P, Domingues MRM, Freire C, Cardoso SM, Vilas-Boas M. Phenolic Profiling of Portuguese Propolis by LC–MS Spectrometry: Uncommon Propolis Rich in Flavonoid Glycosides. *Phytochemical Analysis* (2013) 24:309–318. doi: 10.1002/pca.2412
25. Kamali Sarvestani Afrand. *Metabolomic profiling of lignocellulosic biomass process streams*. (2016). 133 p.
